# Phenotypic and functional characterization of soil *Pseudomonas* strains reveals multi-metal tolerance and bioremediation potential

**DOI:** 10.3389/fmicb.2025.1731818

**Published:** 2025-12-17

**Authors:** Alessandro De Santis, Antonio Bevilacqua, Sara Conceição, Marta Laranjo, Matteo Francavilla, Mauro Marone, Maria Rosaria Corbo, Milena Sinigaglia

**Affiliations:** 1 Department of Agriculture, Food, Natural Resources and Engineering, University of Foggia, Foggia, Italy; 2 MED-Mediterranean Institute for Agriculture, Environment and Development and CHANGE-Global Change and Sustainability Institute, IIFA-Institute for Advanced Studies and Research, Polo da Mitra da Universidade de Évora, Évora, Portugal; 3 Departamento de Medicina Veterinária, Escola de Ciências e Tecnologia, Polo da Mitra da Universidade de Évora, Évora, Portugal

**Keywords:** *Pseudomonas*, heavy metal, bioremediation, soil contamination, metal resistance, metal reduction

## Abstract

**Introduction:**

The increasing occurrence of illegal urban waste dumping represents a growing environmental concern due to the accumulation of heavy metals in soils and their long-term impact on soil health and microbial communities.

**Methods:**

This study investigated indigenous *Pseudomonas* isolates from 12 contaminated soils collected in Southern Italy, aiming to evaluate their adaptive responses to abiotic stress and their potential for bioremediation. A total of one hundred isolates were obtained and screened for growth under heavy-metal stress conditions (Cu, Pb, Cr, Zn, As). Growth-based tolerance assays were used as a preliminary screening, and only isolates showing measurable removal capacity were further characterized using ICP–OES analysis.

**Results and discussion:**

Selected strains exhibited broad tolerance across different single-metal exposures, with removal efficiencies exceeding 65% for Cr, Zn, Cu, and As, and up to 90% for Pb. These isolates also demonstrated robust growth under osmotic and acidic stress, and maintained viability at low temperatures, suggesting a high level of ecological adaptability. Phylogenetic analysis based on 16S rRNA sequencing confirmed their affiliation within the *P. fluorescens* and *P. putida* groups, taxa known for their metabolic flexibility and stress resistance. The convergence of high removal efficiency, stress tolerance, and phylogenetic diversity indicates that these isolates represent strong candidates for bioaugmentation in metal-polluted soils. Future studies will focus on co-exposure experiments with mixed metals to evaluate synergistic and antagonistic effects and to validate their performance under complex contamination scenarios.

## 1 Introduction

Environmental pollution caused by the illegal dumping of urban waste has become a critical environmental concern worldwide. The uncontrolled deposition of household, industrial, and construction waste in unauthorized areas introduces persistent pollutants into the environment, particularly heavy metals such as lead (Pb), cadmium (Cd), chromium (Cr), copper (Cu), and zinc (Zn) (Alengebawy et al., 2021; Mitra et al., 2022; Sazykin et al., 2023). Due to their non-biodegradable nature and high potential for bioaccumulation, heavy metals persist in soils and sediments, where they disrupt microbial communities, alter nutrient cycling, and impair soil fertility and ecosystem functioning (Pande et al., 2022; Rashid et al., 2023; Popoola et al., 2024). Their accumulation also represents a risk for human health through the contamination of crops and groundwater resources (Rai et al., 2019; Briffa et al., 2020; Perković et al., 2022).

Traditional physicochemical remediation techniques, such as soil excavation, chemical immobilization, and soil washing, can reduce metal concentrations but are often costly, invasive, and environmentally disruptive (Kumar et al., 2023). In contrast, bioremediation, based on the metabolic capabilities of microorganisms, offers a more sustainable and eco-compatible alternative. Through processes such as biosorption, bioaccumulation, enzymatic reduction, and precipitation, microorganisms can detoxify or immobilize heavy metals while preserving soil functionality (Coelho et al., 2015; Singh et al., 2022).

Among the microbial taxa explored, the genus *Pseudomonas* is of particular interest due to its remarkable metabolic versatility, resistance to oxidative and osmotic stress, and its ability to produce extracellular polymeric substances (EPS) that enhance biofilm formation and metal sequestration (Qurbani et al., 2022; Roy et al., 2024). The presence of multiple efflux systems and metal-resistance operons (e.g., *czc*, *cop*, *cad*, *ars*) allows *Pseudomonas* spp. to survive and adapt in contaminated environments, making them strong candidates for biotechnological applications in soil restoration (Rahman and Singh, 2020). Recent studies have confirmed their high detoxification potential: for example, *Pseudomonas* isolates achieved up to 96% Cr(VI) removal through bioflocculant-assisted biosorption (Monga et al., 2022), 76% Cr(VI) reduction coupled with pyrene degradation via chrA and copZ genes (Su et al., 2022), and over 90% Cd and Ni removal when immobilized on biochar (Manikandan and Nair, 2023). Additional reports demonstrated As(III) oxidation (Satyapal et al., 2024) and plasmid-encoded chromate reduction in *P. putida* (Sundarraj et al., 2023), reinforcing the role of this genus as a model system for heavy-metal bioremediation.

In the past decade, genomic and metagenomic studies have expanded our understanding of *Pseudomonas* adaptability and the genetic basis of metal resistance (Khan et al., 2022). However, these approaches often remain predictive: they describe the genetic potential of microbial communities without validating their actual physiological performance under stress. Many studies isolate only a single highly resistant strain from environmental samples, overlooking other microorganisms that may contribute to detoxification through complementary mechanisms. This bias limits our understanding of how phenotypic diversity and ecological adaptation emerge within native microbial communities exposed to multiple contaminants. For this reason, integrating metagenomic insights with classical culture-based screening is essential to link genotypes to functional traits. Controlled phenotypic assays enable the quantification of tolerance and removal efficiency, offering a direct measure of bioremediation potential.

Illegal dumping sites, chronically exposed to mixed waste streams, represent unique ecological niches where indigenous microorganisms are naturally selected for resilience to complex contamination. These environments constitute valuable reservoirs of strains capable of surviving extreme physicochemical stress and exhibiting relevant detoxification traits (Mishra et al., 2019; Sharma et al., 2022; Bora et al., 2025).

In this context, the present study aims to isolate and characterize indigenous *Pseudomonas* strains from soils affected by illegal urban waste dumping, combining phenotypic and molecular identification with the evaluation of their tolerance and removal capacities toward multiple heavy metals. This work seeks to (i) identify native isolates showing significant resistance and detoxification efficiency; (ii) elucidate physiological traits potentially linked to metal stress adaptation, such as EPS production; and (iii) provide a framework for the selection of promising strains for future bioaugmentation and bioremediation strategies.

## 2 Materials and methods

### 2.1 Sampling context and characteristics

Soil samples were collected from 12 different sites surrounding the city of Foggia (Southern Italy), an area affected by long-standing illegal dumping activities. The sites were georeferenced using WGS84 coordinates (Supplementary Table S1), with precise latitude and longitude recorded for each location. The selection was based on visual indicators of anthropogenic disturbance and the likelihood of harboring microbial populations exposed to metal stress. Samples were collected from the top 0–10 cm of the soil surface using sterile tools, transported to the laboratory under refrigerated conditions, and processed within 24 h. They exhibited neutral to slightly alkaline pH (7.2–7.8), EC 0.55–16.7 dS m^−1^, organic matter 2.1–9.8%, and available P 71–668 mg kg^−1^. Total metal concentrations (mg kg^−1^) were: Pb 45–467, Cd 0.1–1.8, Cr 15–109, Cu 13–1,002, Ni 10–73, Zn 30–6,341, As 3.3–11.1.

### 2.2 Isolation and maintenance of *Pseudomonas* spp.

Soil samples (1 g) were suspended in sterile physiological saline solution (0.9% NaCl) and serially diluted. Aliquots (100 μL) of appropriate dilutions were spread on Pseudomonas Agar Base (PAB) (Oxoid) supplemented with Cetrimide-Fucidin-Cephaloridine (C-F-C) (Oxoid), a selective supplement to inhibit non-*Pseudomonas* bacteria. Plates were incubated aerobically at 25 °C for 48 h. Colonies with distinct *Pseudomonas*-like morphology (mucoid, convex, translucent to opaque, often pigmented) were subcultured on fresh PAB plates and purified through successive streaking.

A total of 100 bacterial isolates presumptively belonging to Pseudomonadaceae were recovered from contaminated soils, with strain identifiers assigned sequentially and mapped to specific sampling sites as follows: site 1 (IDs FG1–FG9), site 2 (FG10–FG19), site 3 (FG20–FG27), and so forth, up to site 12 (FG92–100), as described in Supplementary Table S1. To improve readability, the code used in figures will take into account just the number of the code. Purified isolates were maintained on Nutrient agar (Oxoid, Basingstoke, UK) slants at 4 °C for short-term use and preserved in glycerol stocks at −20 °C for long-term storage. These isolates were later subjected to morphological, biochemical, and functional screening to assess their ecological and bioremediative potential.

### 2.3 Morphological and biochemical characterization of *Pseudomonas* isolates

The morphology of the colonies that was observed on PAB was used for evaluation of pigment production, surface texture, elevation, edge definition, and opacity. Gram staining was performed on fresh cultures, after 24 h incubation at optimum temperature, to confirm Gram-negative cell structure and rod-shaped morphology. Furthermore, a series of key biochemical assays were conducted to assess the metabolic functionality of the isolates. Catalase activity was evaluated based on the formation of oxygen bubbles upon exposure to 3% hydrogen peroxide, indicating the presence of the catalase enzyme (Whittenbury, 1964). Oxidase activity (Steel, 1961) was determined using commercial oxidase reagent strips (Merck Millipore, Darmstadt, Germany), allowing for the detection of cytochrome c oxidase. The oxidative or fermentative metabolism of glucose was assessed through the oxidative/fermentative (O/F) test (Porres and Stanyon, 1974), performed in Hugh and Leifson’s medium (Biolife, Milan, Italy). Finally, bacterial motility was investigated using a semi-solid agar medium (0.4% w/v) (Tittsler and Sandholzer, 1936), enabling the observation of active cellular movement through diffuse growth patterns within the medium.

### 2.4 Functional phenotypical characterization

#### 2.4.1 Growth index assessment under pH and temperature stress

Overnight cultures of each isolate were grown in nutrient broth (Oxoid, Basingstoke, UK) and subsequently inoculated at a concentration of 5–6 log CFU/mL into 5 mL of fresh nutrient broth. Prior to inoculation, the medium was adjusted to specific environmental conditions to evaluate the physiological adaptability of the strains. In particular, the pH of the broth was modified to 4.5, 5.5, 6.5 (used as the control), 7.5, and 8.5, using sterile 1 M HCl or 1 M NaOH prior to autoclaving and verified with a calibrated pH, while temperature conditions were set at 15 °C, 25 °C (control), and 37 °C.

Cultures were incubated under agitation (150 rpm) for 24 and 48 h. The optical density at 600 nm (OD₆₀₀) was measured at both times using a UV–Vis spectrophotometer.

The Growth Index (GI) was calculated for each condition as described previosuly (Bevilacqua et al., 2009), using the following Equation 1:


$$\mathrm{GI}=({\mathrm{Abs}}_{\text{sample}}/{\mathrm{Abs}}_{\text{control}})\times 100$$ (1)

Where Abs_sample corresponds to the OD₆₀₀ under stress conditions and Abs_control_ is the OD₆₀₀ under optimal conditions (pH 6.5 and 25 °C).

#### 2.4.2 Drought tolerance simulation

Osmotic stress resistance was tested in nutrient broth supplemented with polyethylene glycol (PEG 6000) (Sigma-Aldrich, Darmstadt, Germany) at final concentrations of 0% (control), 5, 10, 15, and 20%. Isolates were incubated for 48 h at 25 °C, and growth was assessed spectrophotometrically (OD₆₀₀). The drought resistance (DR) was determined by comparing the samples’ OD_600_ against the control by the Equation 2:


$$\mathrm{DR}=({\mathrm{Abs}}_{\text{sample}}/{\mathrm{Abs}}_{\text{control}})\times 100$$ (2)

### 2.5 Heavy metal resistance assays

#### 2.5.1 Growth assay in liquid medium

Minimum Inhibitory Concentration (MIC) assays were performed as a preliminary qualitative screening to identify isolates capable of sustaining growth under increasing metal concentrations. This step was used to reduce the initial collection to the most resistant isolates, which were subsequently tested for quantitative removal capacity. The isolates were exposed to zinc sulfate (ZnSO₄·7H₂O), lead nitrate (Pb(NO₃)₂), potassium dichromate (K₂Cr₂O₇), copper sulfate (CuSO₄·5H₂O), cadmium chloride (CdCl₂·2.5H₂O) and sodium arsenite (NaAsO₂). All 100 isolates were initially inoculated into 10 mL of nutrient broth containing 100 mg/L, 200 mg/L or 300 mg/L of a heavy metal. Cultures were prepared from overnight metal-free precultures and standardized to an initial OD₆₀₀ of approximately 0.05. Incubation was carried out at 28 °C with shaking at 150 rpm for 48 h. At the end of the incubation period, bacterial growth was quantified by measuring optical density at 600 nm (OD₆₀₀). All assays included a zero-metal control prepared with the same medium without heavy-metal supplementation. Data were modeled as Growth Index, using the zero-metal samples as references.

#### 2.5.2 Removal assay in liquid medium

To evaluate the heavy metal removal potential of *Pseudomonas*, isolates that had shown the highest resistance in preliminary assays were selected. Test concentrations were 200 mg/L for Cr and Zn, and 300 mg/L for Cu, Pb, As. Cd removal was not assessed because no isolates survive to more than 100 mg/L.

Each isolate was inoculated at a concentration of 5–6 log CFU/mL into 5 mL of nutrient broth supplemented with specific concentrations of selected heavy metal salts, to evaluate their tolerance thresholds. Cultures were incubated in contaminated nutrient broth for 48 h at 25 °C with shaking at 150 rpm. After incubation, samples were centrifuged at 5000 × g for 10 min to separate the biomass. The supernatant was collected and subjected to organic matter digestion to eliminate potential interference prior to metal quantification.

The residual metal concentrations in digested broth were determined by Inductively Coupled Plasma Optical Emission Spectroscopy (ICP-OES, Agilent 5,800) following acid digestion according to ISO 14870:2001 (International Organization for Standardization, 2001). Samples were mineralized in a microwave oven (MARS6-CEM) using a nitric acid–hydrogen peroxide- hydrofluoridric acid solution (20:7.5:2.5 volume ratio) in accordance with standard protocols for trace metal analysis in biological matrices. Metals quantification was perfomed using a certified multi-standard (Periodic table mix1 for ICP, Supelco).

The removal efficiency (RE%) for each metal was calculated using the formula:


$$\mathrm{RE}\;(\%)=((C_{0}-C_{x})/C_{0})\times 100$$

Where:

- C_0_ = initial metal concentration (mg/L)
- C_x_ = residual metal concentration after incubation (mg/L)

Uninoculated control samples containing only the metal-supplemented medium were included to account for abiotic losses.

### 2.6 Molecular identification of bacterial isolates

Genomic DNA was extracted from 24-h cultures grown in nutrient broth using the E. Z. N. A.® Bacterial DNA Kit (Omega Bio-Tek, USA), according to the manufacturer’s instructions and stored at −20 °C until use.

The 16S rRNA gene was amplified using the forward primer Y1 (5′-TGGCTCAGAACGAACGCTGGCGGC-3′) (Young et al., 1991) and the reverse primer Y3 (5′-TACCTTGTTACGACTTCACCCCAGTC-3′) (Laranjo et al., 2004). Each reaction was prepared in a final volume of 50 μL, consisting of: 34.25 μL nuclease-free water (NZYTech, Portugal), 5 μL 10 × buffer (NZYTech), 2.5 μL MgCl₂ (50 mM) (NZYTech), 1 μL dNTP (10 mM) mix (NZYTech), 3 μL of each primer 5 pmol/μL, 0.25 μL Taq polymerase (5 U/μL) (NZYTech), 1 μL of DNA template (NZYTech).

Polymerase Chain Reaction (PCR) was carried out using a Bio-Rad MJ Mini thermal cycler, following a standardized amplification protocol. The cycling conditions included an initial denaturation at 95 °C for 3 min, followed by 25 cycles (denaturation at 95 °C for 30 s, annealing at 62 °C for 30 s, extension at 72 °C for 90 s) and a final extension at 72 °C for 10 min. PCR products were visualized by agarose gel electrophoresis. Each PCR product (1 μL) was mixed with 2 μL of GelRed and 2 μL Gel Loading Solution (GLS) (0.25% bromophenol blue, 30% glycerol) and then examined under UV transillumination. Amplicons showing a clear band of expected size, against an appropriate molecular weight marker (Ladder III), were purified using ExoCleanUp FAST (VWR, Denmark), according to the manufacturer instructions. Purified products were stored at −20 °C until Sanger sequencing. Raw chromatograms were trimmed, and consensus FASTA sequences were assembled and inspected in Bioedit (v. 7.7.1) (Laranjo et al., 2008). After quality filtering and trimming, a final alignment of approximately 1,400 bp was obtained for each isolate. Chimera screening was performed using UCHIME (Edgar et al., 2011) implemented in VSEARCH (v. 2.30.0) with default parameters (Rognes et al., 2016). The 16S rRNA sequences obtained were compared with reference sequences retrieved from the NCBI GenBank database. Phylogenetic reconstruction was performed in MEGA v.12 (Kumar et al., 2024) using MUSCLE as aligning algorithm and the Maximum Likelihood (ML) method under Hasegawa–Kishino–Yano model with a proportion of invariable sites (HKY + I) (Hasegawa et al., 1985; Luo et al., 2010), selected as the best-fit model based on the lowest Bayesian Information Criterion (BIC) score. Gaps and missing data were handled with the partial deletion (95% site coverage) option. The robustness of the resulting topology was assessed by bootstrap analysis with 1,000 replicates (Laranjo et al., 2012). *Escherichia coli* (accession number X80725.1) and *Aeromonas media* (accession number X60410.2) were included as outgroup sequences.

### 2.7 Statistics

All experiments were performed at least in duplicate. Preliminary assays, including minimum inhibitory concentration (MIC) screening and morphological characterization on PAB, were conducted in duplicate to verify reproducibility. Growth index measurements under temperature, pH, and osmotic stress, as well as metal removal assays determined by ICP–OES, were performed in triplicate to allow statistical evaluation. Data from triplicate experiments were analyzed using STATISTICA software version 12 (StatSoft Inc., Tulsa, OK, USA). Significant differences between groups were assessed using paired t-tests for two-group comparisons or one-way analysis of variance (ANOVA) followed by Tukey’s HSD post-hoc test. For duplicate datasets, only descriptive statistics (mean ± standard deviation) were performed. Differences were considered statistically significant at *p* < 0.05.

## 3 Results

### 3.1 Morphological and biochemical characterization of *Pseudomonas* isolates

A total of 100 presumptive *Pseudomonas* isolates, previously recovered from polluted soils, were screened for multiple functional traits related to bioremediation capacity. All isolates were confirmed as Gram-negative, consistent with the taxonomic characteristics of the genus. Biochemical profiling revealed a predominance of oxidase-positive (87%) and catalase-positive (94%) phenotypes, reflecting the presence of active respiratory enzyme systems. Moreover, the strains predominantly exhibited an oxidative metabolism when tested on O/F medium, in line with their aerobic lifestyle.

### 3.2 Growth index under temperature and pH stress conditions

**Figure 1:**
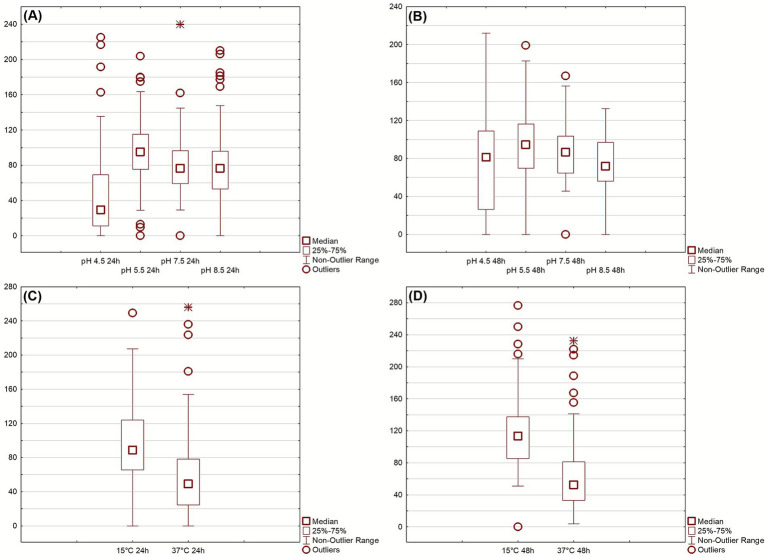
Growth index (GI) of *Pseudomonas* isolates at 24 **(A,C)** and 48 **(B,D)** hours under different stress conditions. Boxplots represent responses to thermal (15 °C, 37 °C) and pH (4.5–8.5) treatments, 25 °C and pH 6.5 used as control. Outliers indicate high-performing or stress-sensitive strains.

**Figure 2:**
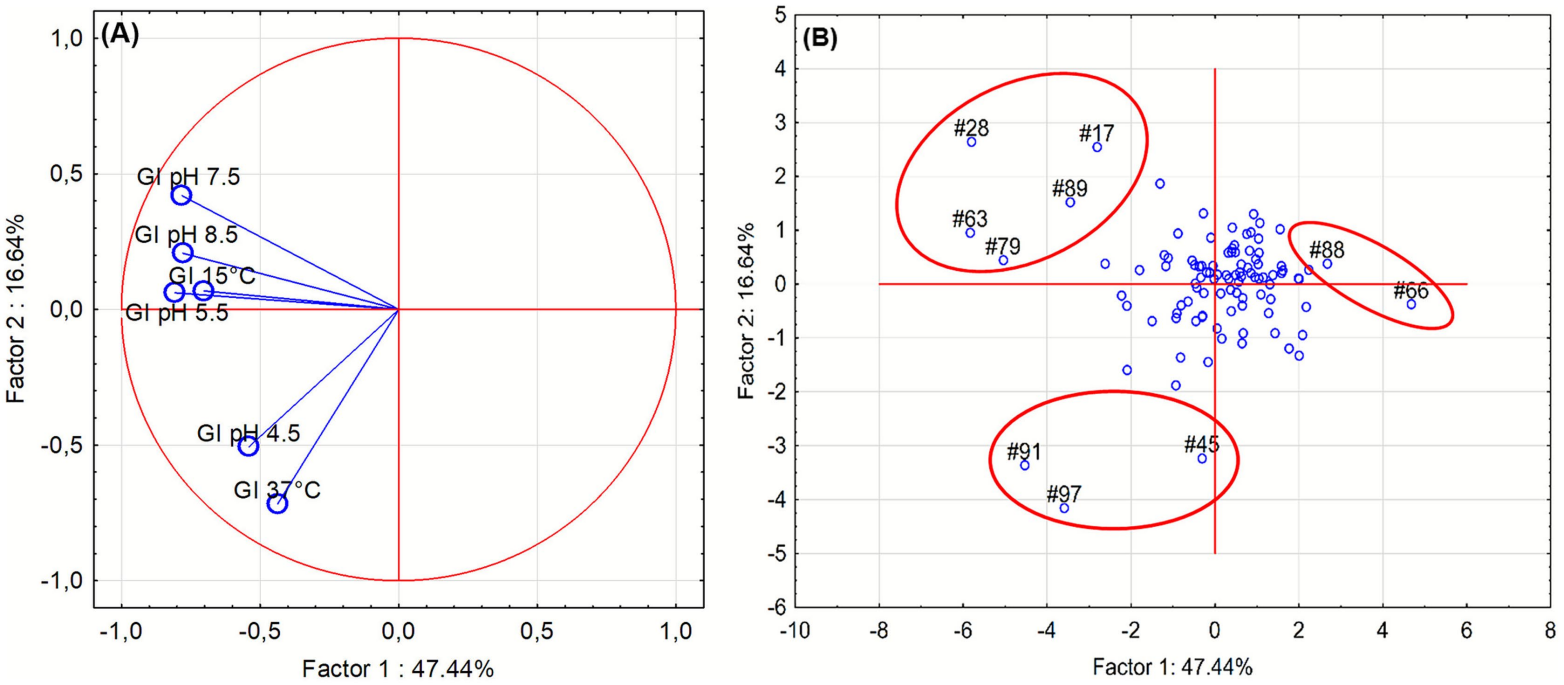
Principal component analysis (PCA) of growth responses of *Pseudomonas* isolates in liquid medium in different stress conditions, after 24 h of incubation. **(A)** Loading plot indicating the contribution of each metal variable to the first two principal components. **(B)** Score plot showing the distribution of isolates.

Growth Index (GI) was evaluated under different abiotic stress conditions at 24 and 48 h (Figure 1). Under acidic conditions, Figures 1A,B, isolates exhibited variable tolerance: growth was markedly reduced at pH 4.5, while it remained stable between pH 5.5 and 8.5, indicating broad pH adaptability with limited acid resistance. Temperature stress Figures 1C,D revealed that most isolates maintained substantial growth at 15 °C but were strongly inhibited at 37 °C, confirming adaptation to moderate rather than elevated temperatures. After 48 h, growth generally improved across all tested conditions, suggesting that most isolates are psychrotolerant and capable of recovering under suboptimal environments. It is worth mentioning that GI approach differs from the normally used method of growth rate and growth kinetic evaluation; in fact, Gi is a “static” measure as it gives a growth performance in some sampling points (for this research 24 and 48 h), and is used by authors as a screening tool to reduce the number of assays in the case of many isolates, as reported elsewhere (Corbo et al., 2017). To summarize the multivariate behavior of the isolates across all tested conditions, a principal component analysis (PCA) was performed using GI values at 24 h. The PCA loading plot (Figure 2A) revealed that Factor 1, which explained 47.44% of the total variance, was primarily influenced by growth at pH 4.5 and 37 °C. Factor 2 (16.64% of variance) was mainly driven by growth at pH 5.5, 7.5, 8.5 and 15 °C.

The PCA score plot (Figure 2B) showed that most isolates clustered near the origin, corresponding to neutral pH and temperature conditions. A few isolates (17, 28, 63, 79, 89) were positioned along the left-upper quadrant, indicating a tendency to better grow in pH 7.5–8.5 and 15 °C conditions. The cluster formed by the isolates 45, 91 and 97 showed a better resistance to acid pH (4.5) and higher temperatures (37 °C) meanwhile the cluster composed by the isolates 66 and 88 appeared at the far right of Factor 1, suggesting low resistance to both acid and thermal stress.

### 3.3 Osmotic stress tolerance under PEG conditions

**Figure 3:**
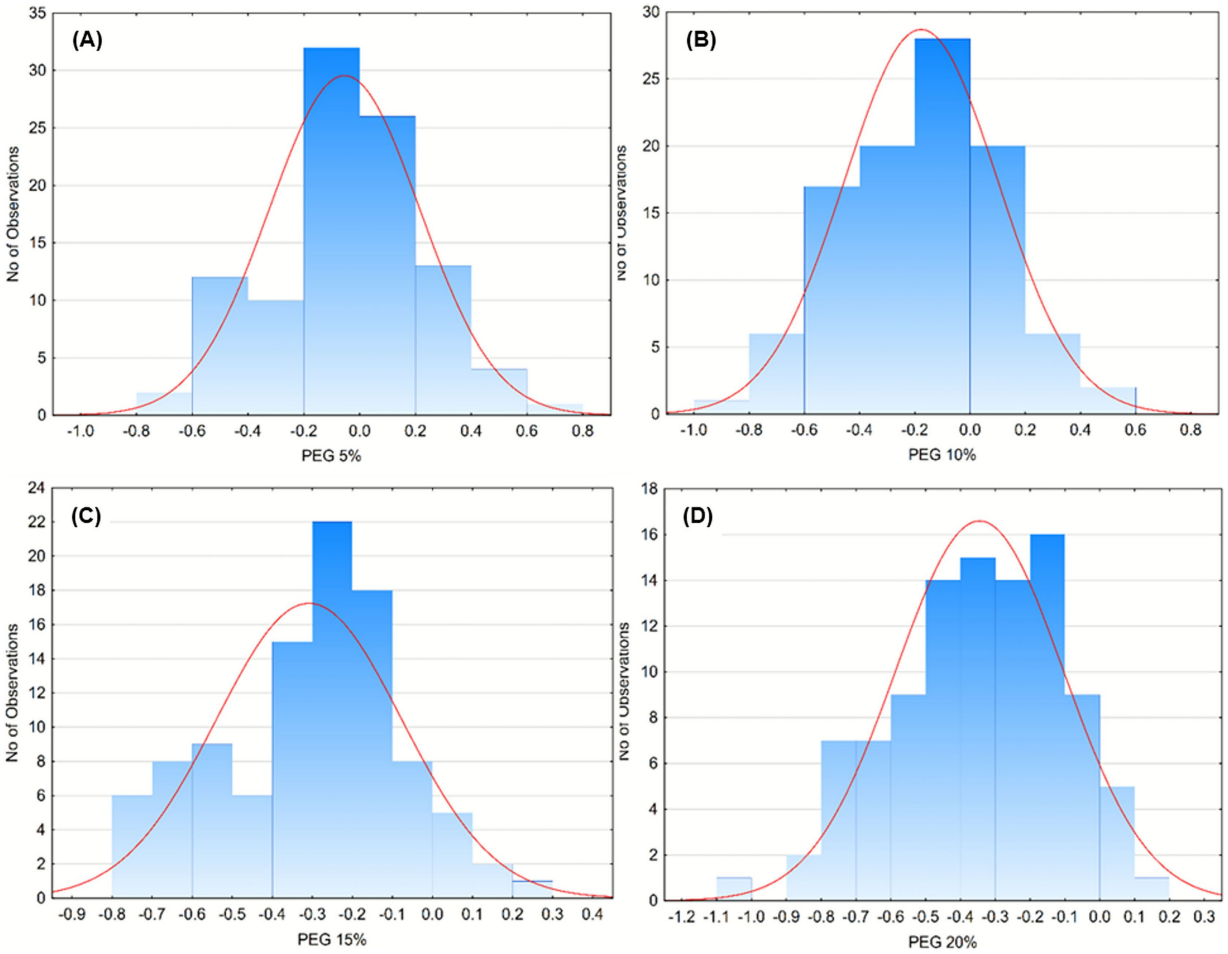
Frequency distribution of bacterial growth responses under increasing concentrations of polyethylene glycol (PEG) as an osmotic stressor. Histograms represent the number of isolates showing specific growth deviations (Y-axis) under PEG concentrations of 5% **(A)**, 10% **(B)**, 15% **(C)**, and 20% **(D)**. The red curve indicates the fitted normal distribution for each dataset. Data illustrate a progressive shift toward more negative growth values with increasing PEG concentration, suggesting enhanced stress sensitivity and reduced tolerance among the microbial isolates.

The tolerance of *Pseudomonas* isolates to osmotic stress was evaluated in media supplemented with polyethylene glycol (PEG) at concentrations of 5, 10, 15, and 20% (Figures 3A–D). Most isolates maintained normal growth at 5% PEG (Figure 3A), showing little to no inhibition compared with control conditions. At 10% PEG (Figure 3B), moderate inhibition appeared in several isolates, although the majority still sustained positive growth indices, suggesting a general capacity to cope with mild osmotic pressure. When PEG concentration increased to 15% (Figure 3C), growth performance markedly declined, with most isolates exhibiting negative GI values and only a limited number maintaining normal growth. At the highest concentration, 20% PEG (Figure 3D), inhibition became widespread, and only a few isolates displayed measurable growth activity, confirming that severe osmotic stress significantly restricts proliferation.

Detailed isolate-specific responses and visual comparisons across PEG concentrations are provided in the Supplementary Materials (Supplementary Figure S1). The heatmap representation allows quick identification of tolerant isolates such as FG36, FG54, FG91, and FG97, which consistently retained neutral or positive growth under high PEG levels, supporting their potential role as robust candidates for application in fluctuating or stress-prone soils.

### 3.4 Growth performance in presence of heavy metals

**Figure 4:**
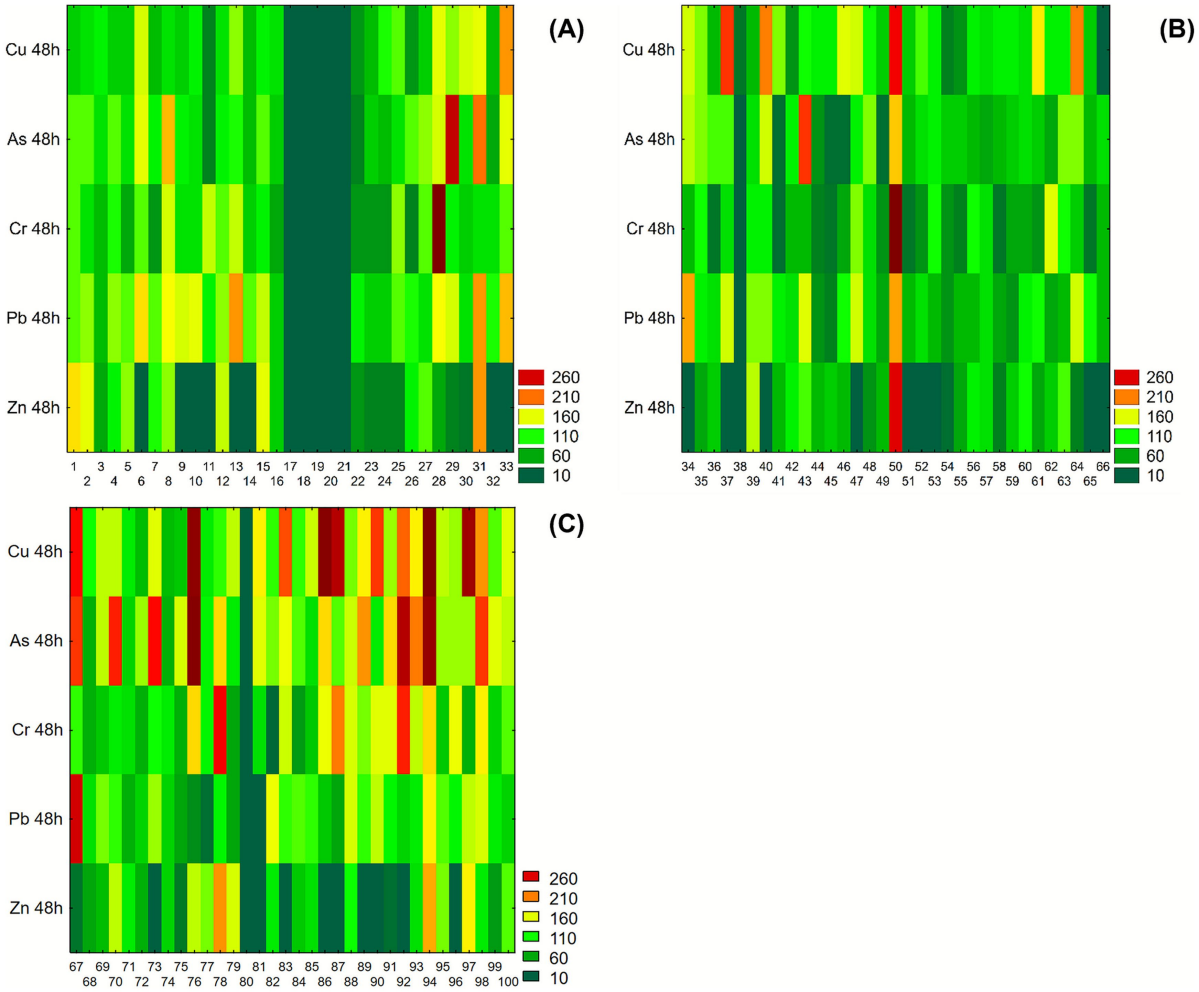
Heatmaps showing growth performance of *Pseudomonas* isolates after 48 h incubation in liquid medium supplemented with 100 mg/L of copper (Cu), arsenic (As), chromium (Cr), lead (Pb), and zinc (Zn). Growth was measured spectrophotometrically and expressed as growth index (GI). Values are color-coded from dark green (low GI) to dark red (high GI). **(A)** Isolates from FG1 to FG33; **(B)** isolates from FG34 to FG66; **(C)** isolates from FG67 to FG100.

The performance of resistance under heavy metals stress (Cu, As, Cr, Pb, Zn, 100 mg/L per each) are resumed in the heatmap representation (Figure 4) after 48 h of incubation in liquid medium supplemented with heavy metals to highlight the different tolerance profiles. In Figure 4A, isolate FG31 displayed consistently good growth or biomass accumulation across Cu, As, Pb, and Zn evident in all corresponding heatmap rows. Isolate FG33 showed a similar trend, particularly under Cu and Pb exposure, while maintaining intermediate growth under Cr and As. Other isolates, such as FG29, stood out for their elevated growth under As stress, showing one of the highest GI values within the dataset. In contrast, isolates from FG17 to FG21 were characterized by low residual growth, under all HMs exposure. In the second group of isolates (from FG34 to FG66), shown in Figure 4B, heterogeneous growth patterns were observed across the five tested metals. Isolate FG50 displayed high GI values in all polluted conditions, suggesting consistent growth in these conditions. Isolates FG37 and FG40 maintained elevated OD levels in Cu, while their response to Zn was comparatively lower. Isolate FG43 showed strong growth in the presence of As, with reduced growth under other metals, indicating a selective response pattern. The remaining isolates in the group displayed mixed and variable behavior, with intermediate to low values more frequently observed under Cu and Pb. In the third group (from FG67 to FG100), represented in Figure 4C, isolate FG94 showed uniformly high growth across all metal conditions, with red intensities evident for Cu, As, Pb, Zn, and Cr. Isolates FG83 and FG89 exhibited elevated GI values particularly under As and Cu, respectively. Isolate FG79 demonstrated high growth in Cu and Cr, while showing moderate values for the remaining metals. Isolate FG74 had its highest growth under As, with lower responses for Zn and Cr. Isolate FG76 displayed poor growth under Zn exposure but maintained high GI levels in Cu and As conditions.

The isolates that exhibited a Growth Index (GI) > 75% after incubation in liquid medium supplemented with 100 mg/L of heavy metals (Zn, Pb, Cr, As, Cu) were subsequently inoculated into medium containing 200 mg/L of the same HM. The same selection criterion (GI > 75%) was then applied to identify candidates for further testing at 300 mg/L. Microorganisms not included in the subsequent tables (Supplementary Tables S2, S3) failed to reach the 75% GI threshold and were therefore excluded from the following steps.

At 200 mg/L (Supplementary Table S2), several *Pseudomonas* isolates retained growth capacity above the 75% threshold in response to at least one metal. Among these, isolate FG1 displayed a broad tolerance profile, with GI values of 110.9% in Zn, 81.4% in Cr, 89.2% in As, and 139.7% in Cu, although Pb resulted in a lower GI (58.5%). Isolate FG5 exhibited consistent high growth across Zn (82.7%), Pb (84.8%), As (140.8%), and Cu (126.5%), while isolate 4 showed GI values above 100% in Zn (120.7%) and Pb (104.8%), but reduced growth in As (20.5%) and Cr (54.1%).

Isolate 7 stood out for its performance under Pb (141.4%), As (104.9%), and Cu (137.3%), despite lower growth under Zn and Cr. Other isolates (e.g., FG2 and FG15) exceeded the threshold in Cu (108.2 and 91.3%, respectively), with FG2 also performing in Pb (75.7%). These results suggest a subset of microorganisms with stable growth responses across metals, particularly As and Cu.

At 300 mg/L (Supplementary Table S3), overall growth performance declined, yet several isolates retained GI values >75%. Isolate FG5 maintained elevated tolerance to As (108.4%) and moderate growth in Zn (72.0%), while isolate FG7 again showed high GI in Cu (126.5%) and sustained growth in As (85.2%). Strain FG4 exceeded the threshold only in Zn (81.7%).

Beyond these, additional isolates demonstrated specific resistance. Isolate FG28 retained high GI under Zn (89.1%), and FG32 responded well to Cu (91.4%). Isolate FG59 showed tolerance to As (90.9%), while FG94 and FG95 were among the few to remain above threshold in Pb (76.0 and 82.0%, respectively), with FG95 also achieving 98.0% in As.

### 3.5 Evaluation of heavy metal removal

Based on MIC screening, only isolates maintaining growth at or above 200 mg/L were retained for subsequent quantitative analyses. These selected strains were subjected to ICP–OES measurements to verify their metal-removal performance.

While the previously described GI evaluation focused on microbial growth in the presence of heavy metals (HMs), a confirmatory experiment was carried out to assess their actual metal removal capacity. Each isolate was inoculated in liquid medium supplemented with a single metal at defined concentrations: 200 mg/L for chromium and zinc, and 300 mg/L for copper, lead, and arsenic. Residual metal concentrations were quantified via ICP-OES and compared to initial values to estimate removal efficiency.

The results, summarized in Supplementary Table S4, revealed marked variability among isolates in terms of residual concentration of HMs in supernatant, expressed as mg/L. For chromium, isolate FG43 showed the best performance (residual Cr: 56.3 ± 0.14 mg/L), followed closely by isolate FG94 (57.3 ± 0.05 mg/L). In the case of lead, isolate FG27 showed the highest removal (14.7 ± 0.11 mg/L), followed by isolates FG76 (69.9 ± 0.04 mg/L) and FG43 (30.0 ± 0.08 mg/L). Zinc removal was most efficient in strain FG77 (42.1 ± 0.12 mg/L), while for arsenic, isolates FG55 the lowest residual concentrations (0.22 ± 0.08 mg/L). Copper removal was generally modest but isolates FG89 (81.7 ± 0.11 mg/L) and FG94 (87.8 ± 0.07 mg/L) performed better than others.

The HMs removal percentages were calculated relative to initial concentrations (Table 1), and twenty-two best performing isolates were selected. There were three inclusion criteria, based on isolates performance (I) removal of at least 3 HM; (II) removal of at least two HM; (III) > 90% HM removal in at least one metal Several isolates showed broad removal capacity across different single-metal, making them promising candidates for bioremediation.

**Table 1:** Heavy metal removal, expressed as percentage, by selected *Pseudomonas* isolates in polluted liquid medium.

Strain Code	Cr Removal (%)	Zn Removal (%)	Cu Removal (%)	As Removal (%)	Pb Removal (%)
FG1	69.31	76.28	66.33		
FG4	68.51	75.29	66.40	79.81	
FG12		74.99		79.54	
FG15		75.61	66.46	80.23	
FG25				74.71	78.65
FG43	71.88				90.01
FG48	67.28			79.45	
FG55				99.93	
FG57				79.77	89.02
FG61			67.27	79.49	
FG63	69.86		68.55	80.09	
FG73				80.08	82.75
FG76					93.45
FG77		78.96		80.28	
FG78		77.49		79.61	88.23
FG83				80.03	76.71
FG88	70.73			79.71	
FG90				80.59	82.10
FG92	68.02			80.93	80.21
FG94	71.41	68.63	70.73		
FG95		77.87			77.55
FG97		77.84	67.47	80.31	77.29

Isolates performance thresholds are (I) removal of at least 3 HM; (II) removal of at least 2 HM; (III) > 90% HM removal in at least one metal.

Among the top performers, isolate FG4 removed 68.5% of Cr, 75.3% of Zn, 66.4% of Cu, and 79.8% of As, indicating broad-spectrum activity. FG1 showed similar efficiency, particularly for Zn (76.3%), Cu (66.3%), Cr (69.5%), except for arsenic. FG15 was especially effective for As (80.2%), Cu (66.5%), and Zn (75.6%), while FG25 stood out for Pb (78.6%) and Cu (74.8%). Other notable isolates included FG76 (Pb > 90%) and FG55 (As>95 79.2%).

### 3.6 Genomic identification of potential bioremediating *Pseudomonas* spp.

The 16S rRNA gene sequences from 22 isolates identified as promising bioremediation candidates *in vitro* were deposited in GenBank; accession numbers are provided in Supplementary Table S5. BLAST alignment against the NCBI GenBank database revealed sequence identities ranging between 98.7 and 100% with reference strains belonging to the genus *Pseudomonas*, confirming genus-level assignment.

**Figure 5:**
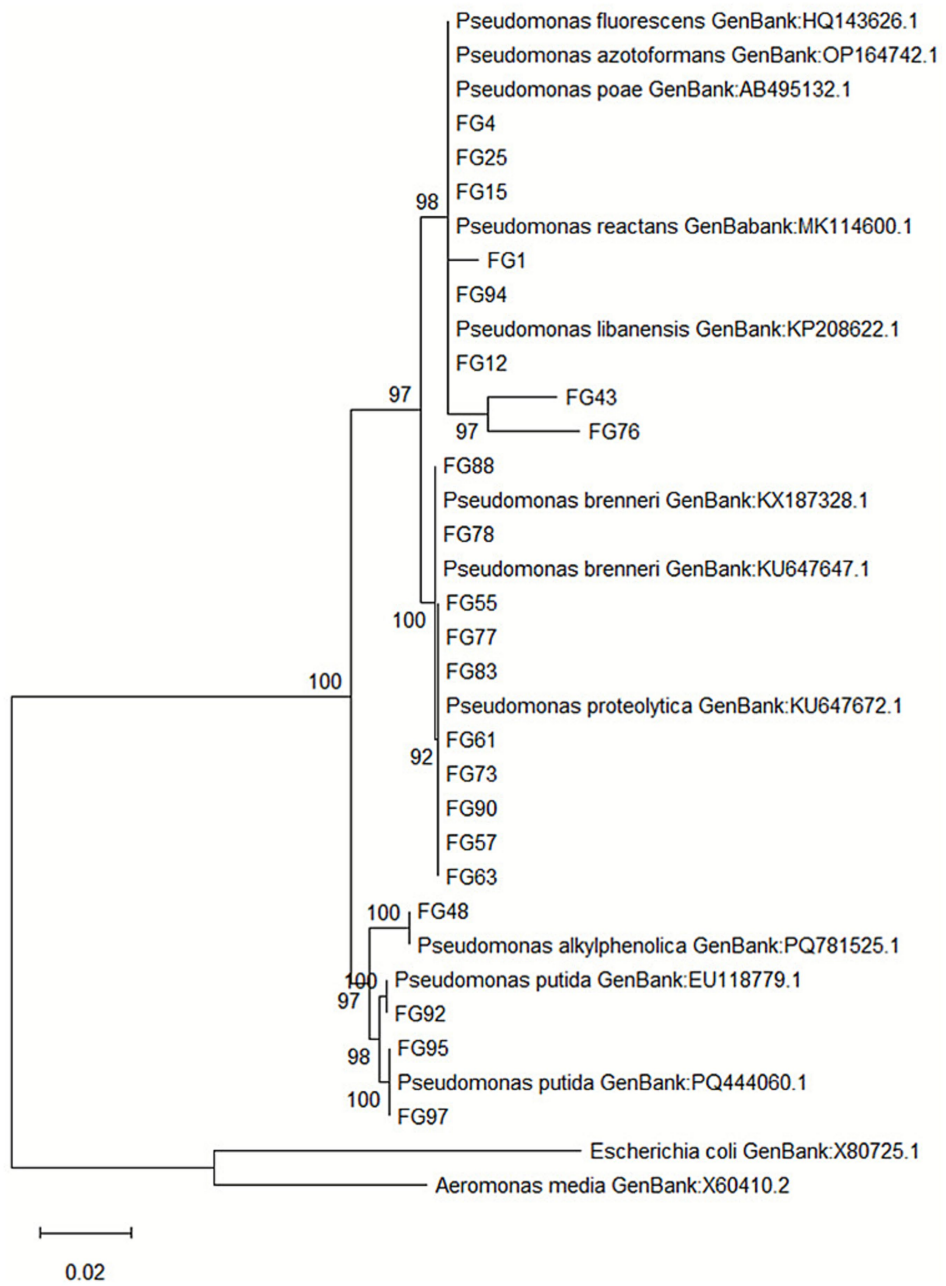
Maximum likelihood phylogenetic tree based on 16S rRNA gene sequences of 22 *Pseudomonas* isolates obtained from polluted soils in the Foggia area (Italy). Sequences were aligned with reference strains retrieved from NCBI GenBank, and evolutionary distances were computed using the Hasegawa, Kishino, and Yano model. Bootstrap values (1,000 replicates) > 90% are shown at the branch nodes. *Escherichia coli* (X80725.1) and *Aeromonas media* (X60410.2) were used as outgroups. Scale bar indicates 0.02 nucleotide substitutions per site.

A phylogenetic tree was constructed using the Maximum Likelihood method with 1,000 bootstrap replicates (Figure 5). The resulting topology resolved the isolates into several well-supported clades (bootstrap ≥92%), consistent with species-level classification.

Cluster I grouped isolates FG4, FG15 and FG25 with *Pseudomonas poae* (AB495132.1) and *Pseudomonas azotoformans* (OP164742.1), with sequence similarity >99%, suggesting their affiliation to the *P. fluorescens* group. FG1 and FG94 clustered tightly with *Pseudomonas reactans* (MK114600.1), sharing 99.1% similarity, while FG12 associated with *P. libanensis* (KP208622.1) at 99.2% identity. Isolates FG43 and FG76 formed a small sub-clade within this group, indicating a close phylogenetic relationship with *P. libanensis* (98.9% similarity) but potentially representing distinct haplotypes. Cluster II included FG88, FG78, FG55, FG77 and FG83, positioned within the *P. brenneri* complex (bootstrap 100, 99.0–99.3% similarity), while FG61, FG63, FG73, FG90 and FG57 grouped with *Pseudomonas proteolytica* (KU647672.1), showing 98.8–99.1% identity. FG48 was closely related to *P. alkylphenolica* (PQ781525.1), sharing 99.3% sequence identity. A distinct and robust clade (bootstrap ≥97%) included FG92, FG95 and FG97, which clustered with *Pseudomonas putida* reference strains (EU118779.1, PQ444060.1), with >99% sequence similarity, confirming their classification as *P. putida*.

## 4 Discussion

This study provides a comprehensive phenotypic and genotypic characterization of *Pseudomonas* isolates from multi-metal contaminated soils, integrating growth performance under physico-chemical stresses, heavy-metal tolerance, quantitative removal capacity, and phylogenetic structure. Microbial communities in polluted soils are continuously subjected to selective pressures such as metal toxicity, oxidative stress, nutrient imbalance, and co-contamination with organic pollutants, which act as evolutionary filters favoring microorganisms equipped with robust detoxification systems, efflux pumps, and regulatory networks for stress adaptation (Qi et al., 2022; Wang et al., 2023; Zou et al., 2024). These conditions may drive microevolutionary divergence within local populations, generating ecotypes that differ not only in tolerance intensity but also in the regulatory balance between stress resistance and metabolic efficiency. *Pseudomonas* spp. is paradigmatic in this scenario, with large genomes, metabolic versatility (Silby et al., 2011), and complex regulons that allow them to survive and function under metal and redox stress (Gillieatt and Coleman, 2024). The isolates characterized here can therefore be interpreted not simply as survivors, but as functionally adapted members of the soil microbiota, actively contributing to natural attenuation and offering potential for bioaugmentation strategies.

Growth Index data confirmed that most isolates are psychrotolerant, sustaining growth at 15 °C and thus ecologically suited for temperate soils, whereas growth was markedly reduced at 37 °C, consistent with the thermal niche of psychrotolerant soil bacteria (Chauhan et al., 2023; Vollmer et al., 2023). Similar observations have been reported for *Pseudomonas* sp. B14-6, a cold-adapted isolate with optimal physiology at low temperatures (Kang et al., 2022), and for *P. fluorescens* strains showing morphological stress at 37 °C (Kahli et al., 2022). This suggests that temperature acts as a stabilizing factor shaping metabolic specialization in these lineages. Psychrotolerance may confer an advantage under field conditions by ensuring enzymatic activity at low temperatures when metal solubility and mobility increase, thus sustaining bioremediation potential in cold or seasonal soils. A subset of strains maintained GI > 80% at pH 4.5, suggesting acid-tolerant ecotypes; this aligns with the well-documented role of pH as a dominant driver of soil community composition, where acidification decreases *α*-diversity and selects for specialized acidophiles (Wang et al., 2022; Hu et al., 2024; Zhang et al., 2024). PEG assays revealed heterogeneous osmotic tolerance, with some isolates retaining normal GI even under high osmotic pressure, an advantageous trait for bioaugmentation in arid or drought-prone soils (Bouremani et al., 2024). This aspect is particularly relevant given that many multi-contaminated soils are marginal or abandoned lands, frequently characterized by poor water retention and seasonal drought. Successful bioaugmentation in such environments requires strains capable of coping with osmotic stress and desiccation, as water scarcity can limit microbial activity and metal immobilization efficiency. Under PEG-imposed stress, superior isolates likely activate sigma-factor (*AlgU*)-mediated regulons controlling membrane stability and stress response (Wang et al., 2024) and induce biosynthesis or uptake of compatible solutes (glycine betaine, proline, ectoine, trehalose) via pathways such as *betB* upregulation (Fan et al., 2024). EPS production and biofilm formation further protect cells by retaining water and buffering local water potential, thereby enhancing persistence under dry or osmotically challenging conditions (Karash and Yahr, 2022; Quan et al., 2022; Chai et al., 2024). It can therefore be hypothesized that osmotic tolerance indirectly contributes to metal resistance, since EPS matrices and compatible solutes also limit ionic toxicity and oxidative stress.

In parallel, heavy-metal exposure imposes oxidative stress, and survival depends on activation of OxyR/SoxR regulons and antioxidant enzymes (catalase, peroxidase, superoxide dismutase), mechanisms recently documented in metal-resistant *Pseudomonas* (Palma et al., 2005; Da Cruz Nizer et al., 2021; Méndez et al., 2022; Pang et al., 2023; Li et al., 2024). MIC screening revealed tolerance up to 300 mg/L for Cu, Pb, and As, consistent with environmental *Pseudomonas* from mining soils (Pramanik et al., 2021; De Santis et al., 2025). Importantly, ICP-OES results confirmed that tolerance translated into actual sequestration: FG4, FG15, FG76, and FG92 removed > 65% of Cr, Zn, Cu, and As, and FG76 achieved > 90% Pb removal, values that place our isolates among the most efficient bioremediators reported in the literature. Growth-based tolerance and metal-removal capacity were intentionally evaluated as distinct traits. MIC data served as preliminary screening to identify viable and resistant isolates, whereas ICP–OES assays provided quantitative evidence of detoxification potential. The absence of a strict linear correlation between MIC and removal efficiency confirms that tolerance and detoxification are governed by partially independent physiological pathways. Highly tolerant isolates likely rely on active efflux and intracellular detoxification, whereas moderately tolerant but high-removal strains depend on surface biosorption and EPS-mediated immobilization.

Comparable efficiencies have been documented for *Pseudomonas* spp. in a variety of systems: for instance, *Pseudomonas stutzeri* LBR achieved significant Pb removal in field soils through bioaugmentation, leading to a measurable decrease in exchangeable Pb fractions and reduced ecological risk indices after treatment (Ridene et al., 2023). In another study, *Pseudomonas alcaliphila* NEWG-2 removed up to 96.6% of Cr(VI) at 200 mg/L using alginate-immobilized cells, with FTIR and SEM–EDS analyses confirming biosorption via carboxyl, hydroxyl, and amide groups and partial bioreduction of Cr(VI) to less toxic Cr(III) (El-Naggar et al., 2020). Similarly, *Pseudomonas veronii* 2E displayed high Cu biosorption capacity (71.4% in 24 h) and maintained performance in continuous packed-bed systems, with successful metal recovery via desorption and potential for process recycling (Busnelli and Vullo, 2022). Together, these studies support the view that the metal removal efficiencies achieved by our isolates are not only quantitatively significant but also mechanistically consistent with established biosorption and bioaccumulation processes observed in high-performance *Pseudomonas* strains. These outcomes are consistent with a composite mechanism involving EPS-mediated biosorption, intracellular bioaccumulation, and microbially induced carbonate precipitation (MICP). It can therefore be hypothesized that FG76 and FG92, characterized by the highest Pb and Cu removals, exploit a combination of EPS-mediated sequestration and MICP processes to achieve rapid and stable immobilization, while FG4 and FG15 may rely more on intracellular accumulation mechanisms supported by membrane-bound reductases. Functional groups within EPS provide abundant cation-binding sites (Balíková et al., 2022), while MICP has been shown to lower Cd bioavailability and is increasingly recognized as a field-viable remediation strategy (Zhang et al., 2023). Beyond physiological mechanisms, genomic determinants such as *czc, cad, cop, ars,* and *mer* operons likely underpin the observed broad-spectrum tolerance, with cross-regulatory interactions (e.g., Czc-Cad systems in *P. putida* KT2440) enhancing multi-metal resilience (Liu et al., 2021; Sánchez-Corona et al., 2025). The phylogenetic reconstruction was performed with the sole aim of confirming the taxonomic affiliation of the isolates within the *Pseudomonas* genus. Given the high intra-specific variability typical of *Pseudomonas*, functional traits such as tolerance and detoxification efficiency are often strain-dependent and cannot be reliably inferred from phylogenetic proximity. The presence of multiple phylogenetic clades among our isolates indicates functional redundancy, a well-recognized stabilizing factor that buffers microbial ecosystem functions under disturbance (Lanari et al., 2022; Chen et al., 2024; Yang et al., 2024; Khidr et al., 2025). This redundancy suggests that metal-removal activity would likely persist even if individual strains were inhibited, consistent with evidence that multi-strain bacterial consortia outperform single isolates for multi-metal bioremediation and enhance system resilience (Khidr et al., 2025; Qattan, 2025). In this perspective, assembling consortia combining tolerant and high-removal isolates could maximize efficiency while minimizing ecological risk, a hypothesis that warrants mesocosm validation. Overall, the convergence of anthropogenic-stress adaptation, psychrotolerance, osmotic and oxidative stress resilience, high MICs with quantitatively verified removals, EPS/MICP-driven immobilization, genomic determinants of resistance, and clade-level redundancy positions FG4, FG15, FG76, and FG92 as excellent candidates for pilot-scale bioaugmentation. Although the tested isolates demonstrated broad tolerance across individual heavy metals, these results do not prove a true multi-metal tolerance under simultaneous exposure. The experiments were performed under single-metal conditions to ensure reproducibility and to disentangle individual metal effects. However, in natural environments, metals often co-occur and may interact additively, antagonistically, or synergistically, influencing both toxicity and detoxification dynamics. Future work will therefore test binary and ternary metal combinations at environmentally relevant ratios to determine whether tolerance and removal capacities are maintained or modified under mixed-metal stress. Such experiments will provide a more realistic evaluation of the ecological performance and biotechnological applicability of the selected strains. Future research should include mechanistic validation (EPS, siderophore, osmolyte, and antioxidant assays; metal speciation), genome-wide screening for resistance loci and ARGs (Sánchez-Corona et al., 2025), design of complementary consortia, mesocosm trials to assess persistence and competitiveness, and immobilization on carriers such as biochar or alginate to maximize survival and operational reusability (Hu et al., 2025; Jhariya et al., 2025).

This study provides an integrated assessment of the adaptive, physiological, and phylogenetic traits of *Pseudomonas* isolates recovered from multi-metal contaminated soils. The combined use of phenotypic screening, metal tolerance assays, and molecular identification enabled the characterization of strains exhibiting diverse stress-response profiles and significant detoxification potential. The results underline the ecological plasticity of *Pseudomonas*, a genus widely recognized for its metabolic versatility, environmental resilience, and ability to persist in complex and contaminated matrices.

The findings support the view that native *Pseudomonas* populations represent an important biological resource for environmentally friendly soil restoration strategies. Their ability to tolerate and remove different metals suggests a promising role in the implementation of bioaugmentation and biostabilization processes aimed at reducing metal mobility and bioavailability. In this perspective, understanding the interplay between tolerance mechanisms, metal-binding structures, and phylogenetic background may facilitate the rational selection of efficient strains and the design of tailored microbial consortia.

Future research should focus on genome-level mapping of resistance operons and co-localized ARGs, detailed metabolite assays (EPS, siderophores, osmolytes, antioxidants), pilot-scale mesocosm trials to assess persistence and competitiveness, and immobilization of selected isolates on carriers such as biochar or alginate to enhance survival, reusability, and operational stability.

## Data Availability

The datasets presented in this study can be found in online repositories. The names of the repository/repositories and accession number(s) can be found in the article/Supplementary material.
