# Supplementary material for: Phenotypic and functional characterization of soil Pseudomonas strains reveals multi-metal tolerance and bioremediation potential
Source: Front Microbiol. 2025 Dec 17;16:1731818. doi: 10.3389/fmicb.2025.1731818 (PMC12754013; doi:10.3389/fmicb.2025.1731818)
Supplement: Supplementary file 1 [file Data_Sheet_1.docx]

# Phenotypic and Functional Characterization of Soil *Pseudomonas* Strains Reveals Multi-Metal Tolerance and Bioremediation Potential

Alessandro De Santis^1^, Antonio Bevilacqua^1^, Sara Conceição^2^, Marta Laranjo^2,3^, Matteo Francavilla^1^, Mauro Marone^1^, Maria Rosaria Corbo^1^, Milena Sinigaglia^1^

^1^Department of Agriculture, Food, Natural Resources and Engineering, University of Foggia, Foggia, ITALY

^2^MED-Mediterranean Institute for Agriculture, Environment and Development & CHANGE-Global Change and Sustainability Institute, IIFA-Institute for Advanced Studies and Research, Universidade de Évora, Pólo da Mitra, Évora, Portugal

^3^ Departamento de Medicina Veterinária, Escola de Ciências e Tecnologia, Universidade de Évora, Pólo da Mitra, Évora, Portugal

*** Correspondence:**Antonio Bevilacqua
antonio.bevilacqua@unifg.it

# Supplementary matherial

**Supplementary Table S1**. Coordinates (WGS84) of the 12 environmental sampling sites in the peri-urban area of Foggia, Italy. Each site is associated with a set of bacterial strains (n = 100), sequentially numbered according to the sampling location.

| **Sampling Site** | **Longitude** | **Latitude** | **Strains**  **(from – to)** |
| --- | --- | --- | --- |
| 1 | 15.585849 | 41.422099 | FG1-FG9 |
| 2 | 15.608232 | 41.444830 | FG10-FG19 |
| 3 | 15.604090 | 41.466206 | FG20-FG27 |
| 4 | 15.584307 | 41.477667 | FG28-FG35 |
| 5 | 15.545304 | 41.477356 | FG36-FG43 |
| 6 | 15.528977 | 41.482316 | FG44-FG51 |
| 7 | 15.492058 | 41.474061 | FG52-FG59 |
| 8 | 15.496651 | 41.452132 | FG60-FG66 |
| 9 | 15.505415 | 41.433294 | FG67-FG73 |
| 10 | 15.508348 | 41.413625 | FG74-FG82 |
| 11 | 15.535685 | 41.421741 | FG83-FG91 |
| 12 | 15.523417 | 41.370251 | FG92-FG100 |

**Supplementary table S2.** Growth Index (GI, %) of *Pseudomonas* isolates after 48 h of incubation in liquid medium supplemented with 200mg/L of heavy metals: zinc (Zn), lead (Pb), chromium (Cr), arsenic (As), and copper (Cu).

| **Zn** | | | | | **Pb** | | | | | **Cr** | | | | | **As** | | | | | **Cu** | | | | |
| --- | --- | --- | --- | --- | --- | --- | --- | --- | --- | --- | --- | --- | --- | --- | --- | --- | --- | --- | --- | --- | --- | --- | --- | --- |
| **Strain** | **48h** | **48h II** | **Mean** | **Std. Dev.** | **Strain** | **48h** | **48h II** | **Mean** | **Std. Dev.** | **Strain** | **48h** | **48h II** | **Mean** | **Std. Dev.** | **Strain** | **48h** | **48h II** | **Mean** | **Std. Dev.** | **Strain** | **48h** | **48h II** | **Mean** | **Std. Dev.** |
| 1 | 110.87 | 110.93 | 110.90 | 0.03 | 1 | 58.42 | 58.57 | 58.50 | 0.07 | 1 | 81.25 | 81.48 | 81.37 | 0.12 | 1 | 89.13 | 89.32 | 89.22 | 0.09 | 1 | 139.67 | 139.74 | 139.71 | 0.03 |
| 2 | 43.87 | 44.00 | 43.93 | 0.07 | 2 | 75.68 | 75.80 | 75.74 | 0.06 | 2 | 61.12 | 61.25 | 61.19 | 0.06 | 2 | 58.21 | 58.40 | 58.31 | 0.10 | 2 | 108.11 | 108.29 | 108.20 | 0.09 |
| 4 | 120.63 | 120.74 | 120.69 | 0.05 | 4 | 104.76 | 104.88 | 104.82 | 0.06 | 4 | 53.97 | 54.17 | 54.07 | 0.10 | 3 | 20.40 | 20.57 | 20.49 | 0.08 | 3 | 86.10 | 86.18 | 86.14 | 0.04 |
| 5 | 82.58 | 82.77 | 82.68 | 0.10 | 5 | 84.68 | 84.87 | 84.78 | 0.09 | 5 | 46.25 | 46.33 | 46.29 | 0.04 | 4 | 140.74 | 140.94 | 140.84 | 0.10 | 4 | 126.45 | 126.63 | 126.54 | 0.09 |
| 7 | 52.88 | 52.96 | 52.92 | 0.04 | 6 | 141.33 | 141.49 | 141.41 | 0.08 | 6 | 23.47 | 23.67 | 23.57 | 0.10 | 5 | 104.80 | 104.97 | 104.89 | 0.08 | 5 | 137.24 | 137.45 | 137.34 | 0.11 |
| 8 | 2.02 | 2.23 | 2.13 | 0.11 | 7 | 47.64 | 47.73 | 47.69 | 0.04 | 8 | 42.75 | 42.97 | 42.86 | 0.11 | 6 | 130.10 | 130.23 | 130.17 | 0.06 | 6 | 128.57 | 128.64 | 128.60 | 0.03 |
| 12 | 57.74 | 57.83 | 57.79 | 0.05 | 8 | 52.11 | 52.29 | 52.20 | 0.09 | 10 | 31.08 | 31.26 | 31.17 | 0.09 | 7 | 21.99 | 22.08 | 22.03 | 0.04 | 8 | 53.58 | 53.72 | 53.65 | 0.07 |
| 15 | 63.18 | 63.32 | 63.25 | 0.07 | 9 | 84.02 | 84.10 | 84.06 | 0.04 | 11 | 50.25 | 50.35 | 50.30 | 0.05 | 8 | 27.16 | 27.27 | 27.21 | 0.06 | 9 | 27.40 | 27.46 | 27.43 | 0.03 |
| 20 | 35.11 | 35.22 | 35.16 | 0.06 | 10 | 58.46 | 58.61 | 58.53 | 0.07 | 12 | 21.26 | 21.45 | 21.35 | 0.09 | 9 | 96.35 | 96.48 | 96.41 | 0.07 | 10 | 67.23 | 67.37 | 67.30 | 0.07 |
| 26 | 31.42 | 31.56 | 31.49 | 0.07 | 11 | 81.86 | 82.03 | 81.94 | 0.08 | 13 | 20.63 | 20.72 | 20.68 | 0.04 | 10 | 46.00 | 46.21 | 46.10 | 0.10 | 12 | 18.90 | 19.04 | 18.97 | 0.07 |
| 27 | 33.23 | 33.29 | 33.26 | 0.03 | 12 | 27.03 | 27.28 | 27.16 | 0.12 | 15 | 24.83 | 24.95 | 24.89 | 0.06 | 12 | 85.30 | 85.47 | 85.38 | 0.08 | 13 | 53.57 | 53.66 | 53.61 | 0.04 |
| 28 | 140.68 | 140.89 | 140.79 | 0.10 | 13 | 65.87 | 66.04 | 65.96 | 0.08 | 20 | 2.13 | 2.24 | 2.18 | 0.05 | 13 | 21.30 | 21.48 | 21.39 | 0.09 | 15 | 76.69 | 76.78 | 76.73 | 0.05 |
| 32 | 102.46 | 102.57 | 102.51 | 0.06 | 14 | 78.42 | 78.56 | 78.49 | 0.07 | 27 | 88.00 | 88.11 | 88.06 | 0.06 | 15 | 75.34 | 75.53 | 75.44 | 0.10 | 16 | 62.28 | 62.37 | 62.33 | 0.04 |
| 56 | 45.91 | 46.11 | 46.01 | 0.10 | 15 | 41.89 | 41.97 | 41.93 | 0.04 | 28 | 13.69 | 13.84 | 13.76 | 0.08 | 18 | 59.05 | 59.12 | 59.08 | 0.04 | 20 | 89.06 | 89.18 | 89.12 | 0.06 |
| 57 | 47.76 | 47.90 | 47.83 | 0.07 | 16 | 55.02 | 55.09 | 55.05 | 0.04 | 30 | 12.93 | 13.16 | 13.04 | 0.11 | 20 | 95.29 | 95.51 | 95.40 | 0.11 | 23 | 57.83 | 58.01 | 57.92 | 0.09 |
| 59 | 132.30 | 132.46 | 132.38 | 0.08 | 18 | 27.75 | 27.94 | 27.84 | 0.09 | 31 | 33.46 | 33.59 | 33.52 | 0.06 | 23 | 58.67 | 58.76 | 58.72 | 0.04 | 24 | 55.13 | 55.31 | 55.22 | 0.09 |
| 60 | 20.00 | 20.12 | 20.06 | 0.06 | 19 | 44.75 | 44.89 | 44.82 | 0.07 | 33 | 23.06 | 23.29 | 23.17 | 0.11 | 24 | 62.30 | 62.44 | 62.37 | 0.07 | 25 | 74.31 | 74.41 | 74.36 | 0.05 |
| 62 | 16.17 | 16.34 | 16.25 | 0.09 | 20 | 75.99 | 76.06 | 76.03 | 0.04 | 34 | 33.07 | 33.28 | 33.18 | 0.11 | 25 | 72.05 | 72.19 | 72.12 | 0.07 | 27 | 26.77 | 26.92 | 26.85 | 0.08 |
| 70 | 32.42 | 32.63 | 32.52 | 0.10 | 22 | 109.09 | 109.19 | 109.14 | 0.05 | 35 | 22.54 | 22.63 | 22.59 | 0.04 | 26 | 46.01 | 46.20 | 46.10 | 0.10 | 28 | 40.68 | 40.93 | 40.81 | 0.12 |
| 77 | 76.47 | 76.66 | 76.57 | 0.09 | 25 | 99.84 | 99.96 | 99.90 | 0.06 | 39 | 77.78 | 77.88 | 77.83 | 0.05 | 27 | 87.38 | 87.56 | 87.47 | 0.09 | 29 | 46.42 | 46.62 | 46.52 | 0.10 |
| 78 | 93.55 | 93.76 | 93.65 | 0.11 | 26 | 69.79 | 70.00 | 69.90 | 0.10 | 40 | 5.88 | 5.97 | 5.93 | 0.04 | 28 | 26.62 | 26.86 | 26.74 | 0.12 | 30 | 4.16 | 4.31 | 4.23 | 0.08 |
| 93 | 51.31 | 51.49 | 51.40 | 0.09 | 27 | 102.46 | 102.62 | 102.54 | 0.08 | 41 | 10.41 | 10.61 | 10.51 | 0.10 | 29 | 150.51 | 150.74 | 150.62 | 0.11 | 31 | 103.61 | 103.71 | 103.66 | 0.05 |
| 94 | 60.15 | 60.21 | 60.18 | 0.03 | 28 | 86.31 | 86.41 | 86.36 | 0.05 | 43 | 55.49 | 55.71 | 55.60 | 0.11 | 30 | 42.73 | 42.87 | 42.80 | 0.07 | 33 | 78.31 | 78.38 | 78.35 | 0.03 |
| 95 | 87.55 | 87.76 | 87.65 | 0.10 | 29 | 45.39 | 45.48 | 45.44 | 0.04 | 47 | 34.85 | 34.91 | 34.88 | 0.03 | 31 | 70.72 | 70.84 | 70.78 | 0.06 | 35 | 73.66 | 73.80 | 73.73 | 0.07 |
| 97 | 148.11 | 148.26 | 148.19 | 0.08 | 30 | 40.18 | 40.43 | 40.31 | 0.12 | 48 | 91.03 | 91.21 | 91.12 | 0.09 | 33 | 60.73 | 60.96 | 60.85 | 0.12 | 36 | 125.92 | 126.03 | 125.97 | 0.06 |
|  |  |  |  |  | 31 | 34.79 | 35.02 | 34.91 | 0.12 | 53 | 15.63 | 15.74 | 15.68 | 0.06 | 34 | 88.33 | 88.43 | 88.38 | 0.05 | 40 | 25.21 | 25.33 | 25.27 | 0.06 |
|  |  |  |  |  | 32 | 85.26 | 85.47 | 85.37 | 0.11 | 56 | 5.28 | 5.37 | 5.32 | 0.05 | 35 | 43.30 | 43.41 | 43.36 | 0.05 | 41 | 136.16 | 136.37 | 136.27 | 0.10 |
|  |  |  |  |  | 33 | 85.16 | 85.25 | 85.21 | 0.05 | 57 | 3.59 | 3.76 | 3.67 | 0.08 | 36 | 125.46 | 125.67 | 125.57 | 0.11 | 43 | 5.27 | 5.48 | 5.38 | 0.10 |
|  |  |  |  |  | 34 | 95.72 | 95.96 | 95.84 | 0.12 | 62 | 46.24 | 46.49 | 46.36 | 0.12 | 37 | 77.32 | 77.52 | 77.42 | 0.10 | 46 | 77.86 | 78.02 | 77.94 | 0.08 |
|  |  |  |  |  | 35 | 39.06 | 39.24 | 39.15 | 0.09 | 63 | 85.92 | 86.00 | 85.96 | 0.04 | 39 | 61.46 | 61.66 | 61.56 | 0.10 | 47 | 83.30 | 83.48 | 83.39 | 0.09 |
|  |  |  |  |  | 36 | 27.98 | 28.12 | 28.05 | 0.07 | 76 | 15.38 | 15.45 | 15.42 | 0.03 | 40 | 42.30 | 42.49 | 42.39 | 0.10 | 48 | 110.76 | 110.95 | 110.85 | 0.09 |
|  |  |  |  |  | 37 | 51.76 | 51.98 | 51.87 | 0.11 | 78 | 46.24 | 46.40 | 46.32 | 0.08 | 42 | 35.00 | 35.16 | 35.08 | 0.08 | 50 | 105.82 | 106.01 | 105.92 | 0.10 |
|  |  |  |  |  | 38 | 86.29 | 86.44 | 86.37 | 0.07 | 83 | 10.46 | 10.59 | 10.53 | 0.07 | 43 | 35.86 | 35.96 | 35.91 | 0.05 | 53 | 168.75 | 168.99 | 168.87 | 0.12 |
|  |  |  |  |  | 39 | 57.64 | 57.75 | 57.69 | 0.06 | 88 | 69.81 | 69.92 | 69.87 | 0.05 | 47 | 12.70 | 12.92 | 12.81 | 0.11 | 56 | 81.00 | 81.10 | 81.05 | 0.05 |
|  |  |  |  |  | 40 | 33.61 | 33.73 | 33.67 | 0.06 | 89 | 6.67 | 6.88 | 6.77 | 0.11 | 48 | 123.77 | 123.86 | 123.81 | 0.05 | 57 | 46.41 | 46.59 | 46.50 | 0.09 |
|  |  |  |  |  | 41 | 68.77 | 68.99 | 68.88 | 0.11 | 90 | 14.43 | 14.61 | 14.52 | 0.09 | 50 | 80.95 | 81.14 | 81.05 | 0.09 | 58 | 175.30 | 175.37 | 175.34 | 0.03 |
|  |  |  |  |  | 42 | 167.50 | 167.70 | 167.60 | 0.10 | 91 | 50.97 | 51.06 | 51.02 | 0.05 | 52 | 60.00 | 60.20 | 60.10 | 0.10 | 59 | 189.49 | 189.67 | 189.58 | 0.09 |
|  |  |  |  |  | 43 | 85.65 | 85.89 | 85.77 | 0.12 | 92 | 90.29 | 90.54 | 90.41 | 0.12 | 53 | 150.78 | 150.96 | 150.87 | 0.09 | 60 | 73.33 | 73.42 | 73.38 | 0.04 |
|  |  |  |  |  | 44 | 25.88 | 26.00 | 25.94 | 0.06 | 93 | 15.36 | 15.43 | 15.39 | 0.03 | 54 | 99.00 | 99.09 | 99.04 | 0.05 | 61 | 148.26 | 148.41 | 148.34 | 0.08 |
|  |  |  |  |  | 46 | 32.63 | 32.72 | 32.68 | 0.04 | 94 | 60.15 | 60.23 | 60.19 | 0.04 | 55 | 171.07 | 171.26 | 171.16 | 0.10 | 62 | 12.78 | 12.88 | 12.83 | 0.05 |
|  |  |  |  |  | 47 | 58.98 | 59.17 | 59.08 | 0.09 | 96 | 8.89 | 9.06 | 8.97 | 0.08 | 56 | 179.16 | 179.23 | 179.19 | 0.04 | 63 | 160.56 | 160.80 | 160.68 | 0.12 |
|  |  |  |  |  | 48 | 60.99 | 61.06 | 61.02 | 0.04 | 98 | 41.67 | 41.86 | 41.76 | 0.10 | 57 | 93.50 | 93.59 | 93.54 | 0.05 | 64 | 12.92 | 13.16 | 13.04 | 0.12 |
|  |  |  |  |  | 50 | 112.70 | 112.88 | 112.79 | 0.09 |  |  |  |  |  | 59 | 106.22 | 106.29 | 106.26 | 0.03 | 65 | 6.57 | 6.67 | 6.62 | 0.05 |
|  |  |  |  |  | 52 | 81.50 | 81.65 | 81.58 | 0.08 |  |  |  |  |  | 60 | 157.04 | 157.28 | 157.16 | 0.12 | 67 | 85.98 | 86.09 | 86.03 | 0.06 |
|  |  |  |  |  | 56 | 84.17 | 84.25 | 84.21 | 0.04 |  |  |  |  |  | 61 | 161.00 | 161.12 | 161.06 | 0.06 | 69 | 122.43 | 122.60 | 122.52 | 0.09 |
|  |  |  |  |  | 57 | 77.80 | 77.89 | 77.84 | 0.04 |  |  |  |  |  | 62 | 81.20 | 81.44 | 81.32 | 0.12 | 70 | 115.93 | 116.06 | 116.00 | 0.07 |
|  |  |  |  |  | 60 | 141.48 | 141.70 | 141.59 | 0.11 |  |  |  |  |  | 63 | 109.39 | 109.60 | 109.50 | 0.11 | 71 | 112.99 | 113.19 | 113.09 | 0.10 |
|  |  |  |  |  | 61 | 98.07 | 98.16 | 98.11 | 0.05 |  |  |  |  |  | 64 | 88.28 | 88.47 | 88.37 | 0.10 | 73 | 6.01 | 6.17 | 6.09 | 0.08 |
|  |  |  |  |  | 64 | 45.22 | 45.44 | 45.33 | 0.11 |  |  |  |  |  | 65 | 60.90 | 61.05 | 60.97 | 0.07 | 76 | 35.63 | 35.75 | 35.69 | 0.06 |
|  |  |  |  |  | 65 | 68.86 | 69.00 | 68.93 | 0.07 |  |  |  |  |  | 66 | 136.57 | 136.80 | 136.69 | 0.12 | 77 | 48.74 | 48.88 | 48.81 | 0.07 |
|  |  |  |  |  | 67 | 104.26 | 104.48 | 104.37 | 0.11 |  |  |  |  |  | 67 | 110.98 | 111.12 | 111.05 | 0.07 | 78 | 159.13 | 159.38 | 159.26 | 0.12 |
|  |  |  |  |  | 69 | 44.16 | 44.34 | 44.25 | 0.09 |  |  |  |  |  | 68 | 69.91 | 70.12 | 70.02 | 0.11 | 79 | 15.26 | 15.33 | 15.30 | 0.03 |
|  |  |  |  |  | 73 | 125.14 | 125.27 | 125.20 | 0.07 |  |  |  |  |  | 69 | 98.83 | 99.03 | 98.93 | 0.10 | 81 | 63.37 | 63.50 | 63.43 | 0.06 |
|  |  |  |  |  | 75 | 97.92 | 98.02 | 97.97 | 0.05 |  |  |  |  |  | 70 | 108.24 | 108.37 | 108.30 | 0.06 | 83 | 131.70 | 131.87 | 131.79 | 0.09 |
|  |  |  |  |  | 76 | 55.87 | 56.11 | 55.99 | 0.12 |  |  |  |  |  | 71 | 134.57 | 134.68 | 134.63 | 0.05 | 85 | 14.71 | 14.82 | 14.77 | 0.05 |
|  |  |  |  |  | 78 | 140.86 | 141.06 | 140.96 | 0.10 |  |  |  |  |  | 72 | 34.29 | 34.49 | 34.39 | 0.10 | 86 | 143.75 | 143.89 | 143.82 | 0.07 |
|  |  |  |  |  | 82 | 50.89 | 50.99 | 50.94 | 0.05 |  |  |  |  |  | 73 | 118.58 | 118.70 | 118.64 | 0.06 | 87 | 114.63 | 114.76 | 114.70 | 0.06 |
|  |  |  |  |  | 83 | 135.29 | 135.38 | 135.34 | 0.05 |  |  |  |  |  | 74 | 84.72 | 84.81 | 84.77 | 0.04 | 89 | 84.85 | 84.93 | 84.89 | 0.04 |
|  |  |  |  |  | 84 | 55.53 | 55.70 | 55.61 | 0.09 |  |  |  |  |  | 75 | 123.61 | 123.75 | 123.68 | 0.07 | 90 | 70.49 | 70.71 | 70.60 | 0.11 |
|  |  |  |  |  | 85 | 145.23 | 145.38 | 145.30 | 0.07 |  |  |  |  |  | 76 | 57.49 | 57.59 | 57.54 | 0.05 | 91 | 9.73 | 9.92 | 9.83 | 0.10 |
|  |  |  |  |  | 86 | 161.87 | 162.07 | 161.97 | 0.10 |  |  |  |  |  | 77 | 101.68 | 101.86 | 101.77 | 0.09 | 92 | 104.85 | 104.99 | 104.92 | 0.07 |
|  |  |  |  |  | 87 | 96.95 | 97.17 | 97.06 | 0.11 |  |  |  |  |  | 78 | 158.06 | 158.13 | 158.10 | 0.03 | 93 | 143.45 | 143.65 | 143.55 | 0.10 |
|  |  |  |  |  | 88 | 93.71 | 93.80 | 93.75 | 0.04 |  |  |  |  |  | 81 | 86.14 | 86.22 | 86.18 | 0.04 | 94 | 125.56 | 125.65 | 125.61 | 0.04 |
|  |  |  |  |  | 89 | 96.97 | 97.09 | 97.03 | 0.06 |  |  |  |  |  | 82 | 52.38 | 52.62 | 52.50 | 0.12 | 96 | 109.52 | 109.59 | 109.55 | 0.03 |
|  |  |  |  |  | 90 | 52.13 | 52.34 | 52.23 | 0.10 |  |  |  |  |  | 83 | 119.61 | 119.86 | 119.73 | 0.12 | 97 | 114.05 | 114.22 | 114.14 | 0.09 |
|  |  |  |  |  | 92 | 135.92 | 136.12 | 136.02 | 0.10 |  |  |  |  |  | 84 | 67.34 | 67.41 | 67.37 | 0.03 | 98 | 59.87 | 59.96 | 59.91 | 0.04 |
|  |  |  |  |  | 94 | 56.02 | 56.13 | 56.07 | 0.06 |  |  |  |  |  | 86 | 120.63 | 120.86 | 120.74 | 0.12 | 100 | 51.88 | 51.99 | 51.93 | 0.05 |
|  |  |  |  |  | 95 | 86.31 | 86.41 | 86.36 | 0.05 |  |  |  |  |  | 88 | 161.01 | 161.21 | 161.11 | 0.10 |  |  |  |  |  |
|  |  |  |  |  | 96 | 96.19 | 96.27 | 96.23 | 0.04 |  |  |  |  |  | 89 | 51.52 | 51.72 | 51.62 | 0.10 |  |  |  |  |  |
|  |  |  |  |  | 97 | 122.70 | 122.86 | 122.78 | 0.08 |  |  |  |  |  | 90 | 120.66 | 120.88 | 120.77 | 0.11 |  |  |  |  |  |
|  |  |  |  |  | 98 | 38.16 | 38.24 | 38.20 | 0.04 |  |  |  |  |  | 91 | 198.44 | 198.51 | 198.48 | 0.03 |  |  |  |  |  |
|  |  |  |  |  |  |  |  |  |  |  |  |  |  |  | 92 | 128.15 | 128.22 | 128.19 | 0.04 |  |  |  |  |  |
|  |  |  |  |  |  |  |  |  |  |  |  |  |  |  | 93 | 110.49 | 110.68 | 110.59 | 0.10 |  |  |  |  |  |
|  |  |  |  |  |  |  |  |  |  |  |  |  |  |  | 94 | 121.43 | 121.49 | 121.46 | 0.03 |  |  |  |  |  |
|  |  |  |  |  |  |  |  |  |  |  |  |  |  |  | 95 | 118.67 | 118.92 | 118.80 | 0.12 |  |  |  |  |  |
|  |  |  |  |  |  |  |  |  |  |  |  |  |  |  | 96 | 56.51 | 56.58 | 56.55 | 0.04 |  |  |  |  |  |
|  |  |  |  |  |  |  |  |  |  |  |  |  |  |  | 97 | 163.24 | 163.38 | 163.31 | 0.07 |  |  |  |  |  |
|  |  |  |  |  |  |  |  |  |  |  |  |  |  |  | 98 | 97.15 | 97.34 | 97.24 | 0.09 |  |  |  |  |  |
|  |  |  |  |  |  |  |  |  |  |  |  |  |  |  | 99 | 115.91 | 116.02 | 115.97 | 0.06 |  |  |  |  |  |
|  |  |  |  |  |  |  |  |  |  |  |  |  |  |  | 100 | 110.82 | 110.96 | 110.89 | 0.07 |  |  |  |  |  |

**Supplementary table S3.** Growth Index (GI, %) of *Pseudomonas* isolates after 48 h of incubation in liquid medium supplemented with 300mg/L of heavy metals: zinc (Zn), lead (Pb), chromium (Cr), arsenic (As), and copper (Cu).

| **Zn** | | | | | **Pb** | | | | | **Cr** | | | | | **As** | | | | | **Cu** | | | | |
| --- | --- | --- | --- | --- | --- | --- | --- | --- | --- | --- | --- | --- | --- | --- | --- | --- | --- | --- | --- | --- | --- | --- | --- | --- |
| **Strain** | **48h** | **48h II** | **Mean** | **Std. Dev.** | **Strain** | **48h** | **48h II** | **Mean** | **Std. Dev.** | **Strain** | **48h** | **48h II** | **Mean** | **Std. Dev.** | **Strain** | **48h** | **48h II** | **Mean** | **Std. Dev.** | **Strain** | **48h** | **48h II** | **Mean** | **Std. Dev.** |
| **1** | 26.56 | 26.69 | 26.63 | 0.06 | **4** | 38.06 | 38.31 | 38.19 | 0.12 | **27** | 51.30 | 51.45 | 51.37 | 0.08 | **1** | 29.24 | 29.37 | 29.30 | 0.06 | **1** | 66.07 | 66.19 | 66.13 | 0.06 |
| **4** | 81.59 | 81.75 | 81.67 | 0.08 | **5** | 57.03 | 57.22 | 57.13 | 0.10 | **63** | 16.03 | 16.16 | 16.09 | 0.06 | **2** | 18.34 | 18.53 | 18.44 | 0.10 | **2** | 45.27 | 45.43 | 45.35 | 0.08 |
| **5** | 71.88 | 72.05 | 71.96 | 0.09 | **6** | 22.75 | 22.98 | 22.86 | 0.12 |  |  |  |  |  | **4** | 108.39 | 108.45 | 108.42 | 0.03 | **3** | 42.50 | 42.57 | 42.54 | 0.04 |
| **7** | 15.37 | 15.49 | 15.43 | 0.06 | **8** | 18.43 | 18.55 | 18.49 | 0.06 |  |  |  |  |  | **5** | 85.16 | 85.27 | 85.21 | 0.05 | **4** | 126.45 | 126.55 | 126.50 | 0.05 |
| **12** | 6.65 | 6.82 | 6.74 | 0.09 | **9** | 51.97 | 52.08 | 52.02 | 0.05 |  |  |  |  |  | **6** | 67.45 | 67.66 | 67.56 | 0.11 | **5** | 78.13 | 78.24 | 78.18 | 0.06 |
| **15** | 36.02 | 36.23 | 36.13 | 0.10 | **10** | 26.06 | 26.29 | 26.17 | 0.12 |  |  |  |  |  | **9** | 33.69 | 33.81 | 33.75 | 0.06 | **10** | 25.87 | 26.08 | 25.97 | 0.10 |
| **28** | 89.12 | 89.01 | 89.07 | 0.05 | **11** | 14.13 | 14.29 | 14.21 | 0.08 |  |  |  |  |  | **12** | 50.36 | 50.48 | 50.42 | 0.06 | **13** | 21.63 | 21.77 | 21.70 | 0.07 |
| **32** | 4.30 | 4.47 | 4.38 | 0.08 | **13** | 28.53 | 28.64 | 28.58 | 0.05 |  |  |  |  |  | **15** | 59.32 | 59.44 | 59.38 | 0.06 | **15** | 91.30 | 91.55 | 91.43 | 0.12 |
| **59** | 67.20 | 67.30 | 67.25 | 0.05 | **14** | 54.46 | 54.52 | 54.49 | 0.03 |  |  |  |  |  | **18** | 90.85 | 90.92 | 90.89 | 0.03 | **20** | 35.85 | 36.08 | 35.97 | 0.11 |
| **77** | 50.72 | 50.78 | 50.75 | 0.03 | **16** | 45.99 | 46.13 | 46.06 | 0.07 |  |  |  |  |  | **20** | 55.93 | 56.07 | 56.00 | 0.07 | **25** | 36.95 | 37.09 | 37.02 | 0.07 |
| **78** | 41.44 | 41.59 | 41.52 | 0.07 | **20** | 6.39 | 6.47 | 6.43 | 0.04 |  |  |  |  |  | **23** | 57.19 | 57.30 | 57.25 | 0.06 | **31** | 18.68 | 18.84 | 18.76 | 0.08 |
| **93** | 28.09 | 28.31 | 28.20 | 0.11 | **22** | 17.77 | 18.00 | 17.88 | 0.12 |  |  |  |  |  | **24** | 72.16 | 72.40 | 72.28 | 0.12 | **36** | 15.51 | 15.61 | 15.56 | 0.05 |
| **94** | 24.94 | 25.19 | 25.07 | 0.12 | **25** | 75.86 | 76.04 | 75.95 | 0.09 |  |  |  |  |  | **25** | 71.59 | 71.76 | 71.68 | 0.08 | **41** | 61.46 | 61.66 | 61.56 | 0.10 |
| **95** | 23.20 | 23.44 | 23.32 | 0.12 | **26** | 81.92 | 82.11 | 82.02 | 0.10 |  |  |  |  |  | **27** | 97.93 | 98.15 | 98.04 | 0.11 | **53** | 7.64 | 7.83 | 7.74 | 0.09 |
| **97** | 141.11 | 141.22 | 141.17 | 0.06 | **27** | 103.11 | 103.31 | 103.21 | 0.10 |  |  |  |  |  | **29** | 35.94 | 36.14 | 36.04 | 0.10 | **56** | 40.51 | 40.59 | 40.55 | 0.04 |
|  |  |  |  |  | **28** | 54.65 | 54.72 | 54.69 | 0.03 |  |  |  |  |  | **31** | 74.54 | 74.70 | 74.62 | 0.08 | **58** | 59.87 | 60.00 | 59.93 | 0.06 |
|  |  |  |  |  | **32** | 52.34 | 52.43 | 52.39 | 0.04 |  |  |  |  |  | **33** | 56.36 | 56.51 | 56.44 | 0.07 | **59** | 48.62 | 48.79 | 48.71 | 0.08 |
|  |  |  |  |  | **33** | 47.27 | 47.45 | 47.36 | 0.09 |  |  |  |  |  | **34** | 41.80 | 42.02 | 41.91 | 0.11 | **60** | 22.02 | 22.23 | 22.12 | 0.10 |
|  |  |  |  |  | **34** | 21.96 | 22.14 | 22.05 | 0.09 |  |  |  |  |  | **36** | 51.51 | 51.66 | 51.58 | 0.08 | **61** | 59.44 | 59.60 | 59.52 | 0.08 |
|  |  |  |  |  | **37** | 46.95 | 47.10 | 47.02 | 0.08 |  |  |  |  |  | **37** | 57.75 | 57.85 | 57.80 | 0.05 | **63** | 76.72 | 76.81 | 76.76 | 0.05 |
|  |  |  |  |  | **39** | 12.77 | 13.00 | 12.89 | 0.12 |  |  |  |  |  | **39** | 39.28 | 39.38 | 39.33 | 0.05 | **69** | 17.94 | 18.07 | 18.01 | 0.06 |
|  |  |  |  |  | **41** | 16.70 | 16.93 | 16.81 | 0.11 |  |  |  |  |  | **42** | 60.07 | 60.19 | 60.13 | 0.06 | **70** | 73.81 | 73.90 | 73.85 | 0.05 |
|  |  |  |  |  | **42** | 37.00 | 37.20 | 37.10 | 0.10 |  |  |  |  |  | **48** | 68.36 | 68.43 | 68.40 | 0.03 | **71** | 44.40 | 44.59 | 44.49 | 0.10 |
|  |  |  |  |  | **43** | 134.48 | 134.72 | 134.60 | 0.12 |  |  |  |  |  | **50** | 90.04 | 90.13 | 90.09 | 0.04 | **89** | 100.40 | 100.56 | 100.48 | 0.08 |
|  |  |  |  |  | **47** | 96.00 | 96.13 | 96.07 | 0.07 |  |  |  |  |  | **52** | 52.63 | 52.78 | 52.71 | 0.08 | **93** | 29.03 | 29.12 | 29.07 | 0.05 |
|  |  |  |  |  | **48** | 22.52 | 22.60 | 22.56 | 0.04 |  |  |  |  |  | **53** | 67.52 | 67.71 | 67.61 | 0.10 | **94** | 63.47 | 63.67 | 63.57 | 0.10 |
|  |  |  |  |  | **50** | 36.36 | 36.58 | 36.47 | 0.11 |  |  |  |  |  | **54** | 90.49 | 90.70 | 90.60 | 0.11 | **97** | 103.33 | 103.53 | 103.43 | 0.11 |
|  |  |  |  |  | **52** | 30.77 | 30.86 | 30.81 | 0.05 |  |  |  |  |  | **55** | 69.96 | 70.13 | 70.05 | 0.09 | **98** | 48.48 | 48.71 | 48.60 | 0.11 |
|  |  |  |  |  | **56** | 73.32 | 73.47 | 73.39 | 0.07 |  |  |  |  |  | **56** | 66.60 | 66.67 | 66.64 | 0.04 | **100** | 27.29 | 27.49 | 27.39 | 0.10 |
|  |  |  |  |  | **57** | 45.68 | 45.90 | 45.79 | 0.11 |  |  |  |  |  | **57** | 69.38 | 69.47 | 69.43 | 0.04 |  |  |  |  |  |
|  |  |  |  |  | **60** | 15.51 | 15.67 | 15.59 | 0.08 |  |  |  |  |  | **59** | 39.22 | 39.34 | 39.28 | 0.06 |  |  |  |  |  |
|  |  |  |  |  | **61** | 41.77 | 41.83 | 41.80 | 0.03 |  |  |  |  |  | **60** | 41.01 | 41.08 | 41.05 | 0.04 |  |  |  |  |  |
|  |  |  |  |  | **65** | 107.95 | 108.01 | 107.98 | 0.03 |  |  |  |  |  | **61** | 125.30 | 125.39 | 125.35 | 0.04 |  |  |  |  |  |
|  |  |  |  |  | **67** | 17.28 | 17.42 | 17.35 | 0.07 |  |  |  |  |  | **62** | 69.25 | 69.43 | 69.34 | 0.09 |  |  |  |  |  |
|  |  |  |  |  | **73** | 71.03 | 71.20 | 71.12 | 0.08 |  |  |  |  |  | **63** | 84.35 | 84.47 | 84.41 | 0.06 |  |  |  |  |  |
|  |  |  |  |  | **75** | 34.40 | 34.49 | 34.45 | 0.05 |  |  |  |  |  | **64** | 57.00 | 57.09 | 57.04 | 0.05 |  |  |  |  |  |
|  |  |  |  |  | **76** | 64.56 | 64.70 | 64.63 | 0.07 |  |  |  |  |  | **65** | 45.48 | 45.56 | 45.52 | 0.04 |  |  |  |  |  |
|  |  |  |  |  | **78** | 90.99 | 91.22 | 91.11 | 0.12 |  |  |  |  |  | **66** | 71.03 | 71.18 | 71.11 | 0.08 |  |  |  |  |  |
|  |  |  |  |  | **82** | 22.63 | 22.73 | 22.68 | 0.05 |  |  |  |  |  | **67** | 53.46 | 53.57 | 53.51 | 0.06 |  |  |  |  |  |
|  |  |  |  |  | **83** | 97.50 | 97.70 | 97.60 | 0.10 |  |  |  |  |  | **68** | 42.37 | 42.60 | 42.49 | 0.11 |  |  |  |  |  |
|  |  |  |  |  | **84** | 65.82 | 65.91 | 65.86 | 0.05 |  |  |  |  |  | **69** | 80.66 | 80.78 | 80.72 | 0.06 |  |  |  |  |  |
|  |  |  |  |  | **85** | 16.09 | 16.34 | 16.22 | 0.12 |  |  |  |  |  | **70** | 155.24 | 155.44 | 155.34 | 0.10 |  |  |  |  |  |
|  |  |  |  |  | **86** | 48.65 | 48.85 | 48.75 | 0.10 |  |  |  |  |  | **71** | 96.92 | 97.15 | 97.04 | 0.12 |  |  |  |  |  |
|  |  |  |  |  | **87** | 30.08 | 30.23 | 30.15 | 0.07 |  |  |  |  |  | **73** | 124.83 | 124.91 | 124.87 | 0.04 |  |  |  |  |  |
|  |  |  |  |  | **89** | 39.36 | 39.48 | 39.42 | 0.06 |  |  |  |  |  | **74** | 62.26 | 62.43 | 62.35 | 0.08 |  |  |  |  |  |
|  |  |  |  |  | **90** | 99.03 | 99.19 | 99.11 | 0.08 |  |  |  |  |  | **75** | 55.96 | 56.13 | 56.05 | 0.08 |  |  |  |  |  |
|  |  |  |  |  | **92** | 136.84 | 136.92 | 136.88 | 0.04 |  |  |  |  |  | **76** | 43.96 | 44.05 | 44.00 | 0.05 |  |  |  |  |  |
|  |  |  |  |  | **94** | 24.72 | 24.94 | 24.83 | 0.11 |  |  |  |  |  | **77** | 55.50 | 55.56 | 55.53 | 0.03 |  |  |  |  |  |
|  |  |  |  |  | **95** | 64.99 | 65.08 | 65.04 | 0.04 |  |  |  |  |  | **78** | 146.85 | 147.03 | 146.94 | 0.09 |  |  |  |  |  |
|  |  |  |  |  | **96** | 57.53 | 57.65 | 57.59 | 0.06 |  |  |  |  |  | **81** | 42.17 | 42.28 | 42.23 | 0.06 |  |  |  |  |  |
|  |  |  |  |  | **97** | 118.33 | 118.57 | 118.45 | 0.12 |  |  |  |  |  | **82** | 37.33 | 37.58 | 37.46 | 0.12 |  |  |  |  |  |
|  |  |  |  |  |  |  |  |  |  |  |  |  |  |  | **83** | 77.71 | 77.85 | 77.78 | 0.07 |  |  |  |  |  |
|  |  |  |  |  |  |  |  |  |  |  |  |  |  |  | **84** | 31.82 | 31.92 | 31.87 | 0.05 |  |  |  |  |  |
|  |  |  |  |  |  |  |  |  |  |  |  |  |  |  | **86** | 67.12 | 67.18 | 67.15 | 0.03 |  |  |  |  |  |
|  |  |  |  |  |  |  |  |  |  |  |  |  |  |  | **88** | 63.99 | 64.05 | 64.02 | 0.03 |  |  |  |  |  |
|  |  |  |  |  |  |  |  |  |  |  |  |  |  |  | **89** | 44.18 | 44.30 | 44.24 | 0.06 |  |  |  |  |  |
|  |  |  |  |  |  |  |  |  |  |  |  |  |  |  | **90** | 121.04 | 121.18 | 121.11 | 0.07 |  |  |  |  |  |
|  |  |  |  |  |  |  |  |  |  |  |  |  |  |  | **91** | 124.33 | 124.41 | 124.37 | 0.04 |  |  |  |  |  |
|  |  |  |  |  |  |  |  |  |  |  |  |  |  |  | **92** | 118.20 | 118.32 | 118.26 | 0.06 |  |  |  |  |  |
|  |  |  |  |  |  |  |  |  |  |  |  |  |  |  | **93** | 40.45 | 40.67 | 40.56 | 0.11 |  |  |  |  |  |
|  |  |  |  |  |  |  |  |  |  |  |  |  |  |  | **94** | 49.00 | 49.18 | 49.09 | 0.09 |  |  |  |  |  |
|  |  |  |  |  |  |  |  |  |  |  |  |  |  |  | **95** | 46.00 | 46.13 | 46.06 | 0.07 |  |  |  |  |  |
|  |  |  |  |  |  |  |  |  |  |  |  |  |  |  | **96** | 30.33 | 30.43 | 30.38 | 0.05 |  |  |  |  |  |
|  |  |  |  |  |  |  |  |  |  |  |  |  |  |  | **97** | 132.22 | 132.42 | 132.32 | 0.10 |  |  |  |  |  |
|  |  |  |  |  |  |  |  |  |  |  |  |  |  |  | **98** | 61.40 | 61.52 | 61.46 | 0.06 |  |  |  |  |  |
|  |  |  |  |  |  |  |  |  |  |  |  |  |  |  | **99** | 50.00 | 50.20 | 50.10 | 0.10 |  |  |  |  |  |
|  |  |  |  |  |  |  |  |  |  |  |  |  |  |  | **100** | 61.23 | 61.31 | 61.27 | 0.04 |  |  |  |  |  |

**Supplementary table S4** Residual concentrations (mg/L) of individual heavy metals measured in culture supernatants after 48 h of incubation with *Pseudomonas* isolates.

| **Cr** | | | **Zn** | | | **Cu** | | | **As** | | | **Pb** | | |
| --- | --- | --- | --- | --- | --- | --- | --- | --- | --- | --- | --- | --- | --- | --- |
| **Sample** | **Mean** | **Std. Dev.** | **Sample** | **Mean** | **Std. Dev.** | **Sample** | **Mean** | **Std. Dev.** | **Sample** | **Mean** | **Std. Dev.** | **Sample** | **Mean** | **Std. Dev.** |
| **1** | 61.42 | 0.04 | **1** | 47.48 | 0.04 | **1** | 101.06 | 0.04 | **4** | 60.62 | 0.04 | **5** | 56.69 | 0.09 |
| **2** | 63.94 | 0.04 | **4** | 49.50 | 0.08 | **4** | 100.90 | 0.10 | **5** | 61.64 | 0.10 | **9** | 60.38 | 0.06 |
| **4** | 63.02 | 0.04 | **5** | 48.42 | 0.14 | **5** | 100.15 | 0.09 | **6** | 59.02 | 0.06 | **14** | 56.74 | 0.04 |
| **11** | 65.11 | 0.03 | **7** | 46.09 | 0.05 | **15** | 100.66 | 0.04 | **12** | 61.50 | 0.12 | **25** | 64.16 | 0.10 |
| **27** | 57.68 | 0.08 | **12** | 50.16 | 0.14 | **41** | 90.45 | 0.09 | **15** | 59.42 | 0.12 | **26** | 43.20 | 0.06 |
| **39** | 61.41 | 0,03 | **15** | 48.88 | 0.10 | **58** | 90.59 | 0.11 | **18** | 61.74 | 0.04 | **27** | 14.69 | 0.11 |
| **43** | 56.38 | 0.14 | **28** | 45.12 | 0.02 | **61** | 98.26 | 0.06 | **20** | 63.19 | 0.07 | **28** | 33.60 | 0.04 |
| **48** | 65.49 | 0.05 | **32** | 47.13 | 0.13 | **63** | 94.43 | 0.09 | **23** | 61.57 | 0.05 | **32** | 46.36 | 0.08 |
| **63** | 60.36 | 0.08 | **59** | 47.48 | 0.08 | **70** | 96.07 | 0.13 | **24** | 65.67 | 0.07 | **43** | 30.04 | 0.08 |
| **88** | 58.63 | 0.09 | **77** | 42.20 | 0.12 | **89** | 81.77 | 0.11 | **25** | 76.01 | 0.13 | **47** | 47.97 | 0.09 |
| **91** | 65.42 | 0.12 | **78** | 45.11 | 0.09 | **94** | 87.87 | 0.07 | **27** | 63.59 | 0.07 | **57** | 33.05 | 0.11 |
| **92** | 63.98 | 0.02 | **93** | 50.52 | 0.14 | **97** | 97.65 | 0.05 | **31** | 62.67 | 0.03 | **65** | 60.56 | 0.06 |
| **94** | 57.30 | 0.12 | **94** | 62.83 | 0.09 |  |  |  | **33** | 62.41 | 0.03 | **73** | 51.86 | 0.12 |
|  |  |  | **95** | 44.39 | 0.13 |  |  |  | **36** | 61.41 | 0.11 | **76** | 19.70 | 0.04 |
|  |  |  | **97** | 44.46 | 0.14 |  |  |  | **37** | 62.61 | 0.09 | **78** | 35.35 | 0.03 |
|  |  |  |  |  |  |  |  |  | **42** | 61.35 | 0.11 | **83** | 69.90 | 0.04 |
|  |  |  |  |  |  |  |  |  | **48** | 61.66 | 0.02 | **84** | 38.55 | 0.07 |
|  |  |  |  |  |  |  |  |  | **50** | 61.47 | 0.07 | **90** | 53.73 | 0.03 |
|  |  |  |  |  |  |  |  |  | **52** | 61.55 | 0.07 | **92** | 59.50 | 0.14 |
|  |  |  |  |  |  |  |  |  | **53** | 62.39 | 0.13 | **95** | 67.40 | 0.06 |
|  |  |  |  |  |  |  |  |  | **54** | 62.75 | 0.13 | **96** | 86.30 | 0.10 |
|  |  |  |  |  |  |  |  |  | **55** | 0.30 | 0.08 | **97** | 68.27 | 0.13 |
|  |  |  |  |  |  |  |  |  | **56** | 60.76 | 0.06 |  |  |  |
|  |  |  |  |  |  |  |  |  | **57** | 61.07 | 0.05 |  |  |  |
|  |  |  |  |  |  |  |  |  | **61** | 61.55 | 0.03 |  |  |  |
|  |  |  |  |  |  |  |  |  | **62** | 60.66 | 0.04 |  |  |  |
|  |  |  |  |  |  |  |  |  | **63** | 59.79 | 0.07 |  |  |  |
|  |  |  |  |  |  |  |  |  | **64** | 57.14 | 0.10 |  |  |  |
|  |  |  |  |  |  |  |  |  | **66** | 57.50 | 0.10 |  |  |  |
|  |  |  |  |  |  |  |  |  | **67** | 60.91 | 0.13 |  |  |  |
|  |  |  |  |  |  |  |  |  | **69** | 60.41 | 0.11 |  |  |  |
|  |  |  |  |  |  |  |  |  | **70** | 59.58 | 0.08 |  |  |  |
|  |  |  |  |  |  |  |  |  | **71** | 60.60 | 0.12 |  |  |  |
|  |  |  |  |  |  |  |  |  | **73** | 59.84 | 0.08 |  |  |  |
|  |  |  |  |  |  |  |  |  | **74** | 61.95 | 0.09 |  |  |  |
|  |  |  |  |  |  |  |  |  | **75** | 62.84 | 0.04 |  |  |  |
|  |  |  |  |  |  |  |  |  | **77** | 59.23 | 0.07 |  |  |  |
|  |  |  |  |  |  |  |  |  | **78** | 61.19 | 0.03 |  |  |  |
|  |  |  |  |  |  |  |  |  | **83** | 59.98 | 0.08 |  |  |  |
|  |  |  |  |  |  |  |  |  | **86** | 58.60 | 0.02 |  |  |  |
|  |  |  |  |  |  |  |  |  | **88** | 60.99 | 0.13 |  |  |  |
|  |  |  |  |  |  |  |  |  | **90** | 58.29 | 0.07 |  |  |  |
|  |  |  |  |  |  |  |  |  | **91** | 55.94 | 0.06 |  |  |  |
|  |  |  |  |  |  |  |  |  | **92** | 57.34 | 0.14 |  |  |  |
|  |  |  |  |  |  |  |  |  | **97** | 59.19 | 0.13 |  |  |  |
|  |  |  |  |  |  |  |  |  | **98** | 60.48 | 0.02 |  |  |  |
|  |  |  |  |  |  |  |  |  | **99** | 59.97 | 0.13 |  |  |  |
|  |  |  |  |  |  |  |  |  | **100** | 54.27 | 0.05 |  |  |  |

**Supplementary table S5**. List of bacterial isolates obtained from contaminated soil samples, with corresponding GenBank accession numbers for 16S rRNA gene sequences.

| **Isolate** | **Accession Number** |
| --- | --- |
| FG1 | PX352722 |
| FG4 | PX352723 |
| FG12 | PX352724 |
| FG15 | PX352725 |
| FG25 | PX352726 |
| FG27 | PX352727 |
| FG28 | PX352728 |
| FG32 | PX352729 |
| FG43 | PX352730 |
| FG48 | PX352731 |
| FG55 | PX352732 |
| FG57 | PX352733 |
| FG61 | PX352734 |
| FG63 | PX352735 |
| FG73 | PX352736 |
| FG76 | PX352737 |
| FG77 | PX352738 |
| FG78 | PX352739 |
| FG83 | PX352740 |
| FG88 | PX352741 |
| FG90 | PX352742 |
| FG91 | PX352743 |
| FG92 | PX352744 |
| FG94 | PX352745 |
| FG95 | PX352746 |
| FG97 | PX352747 |


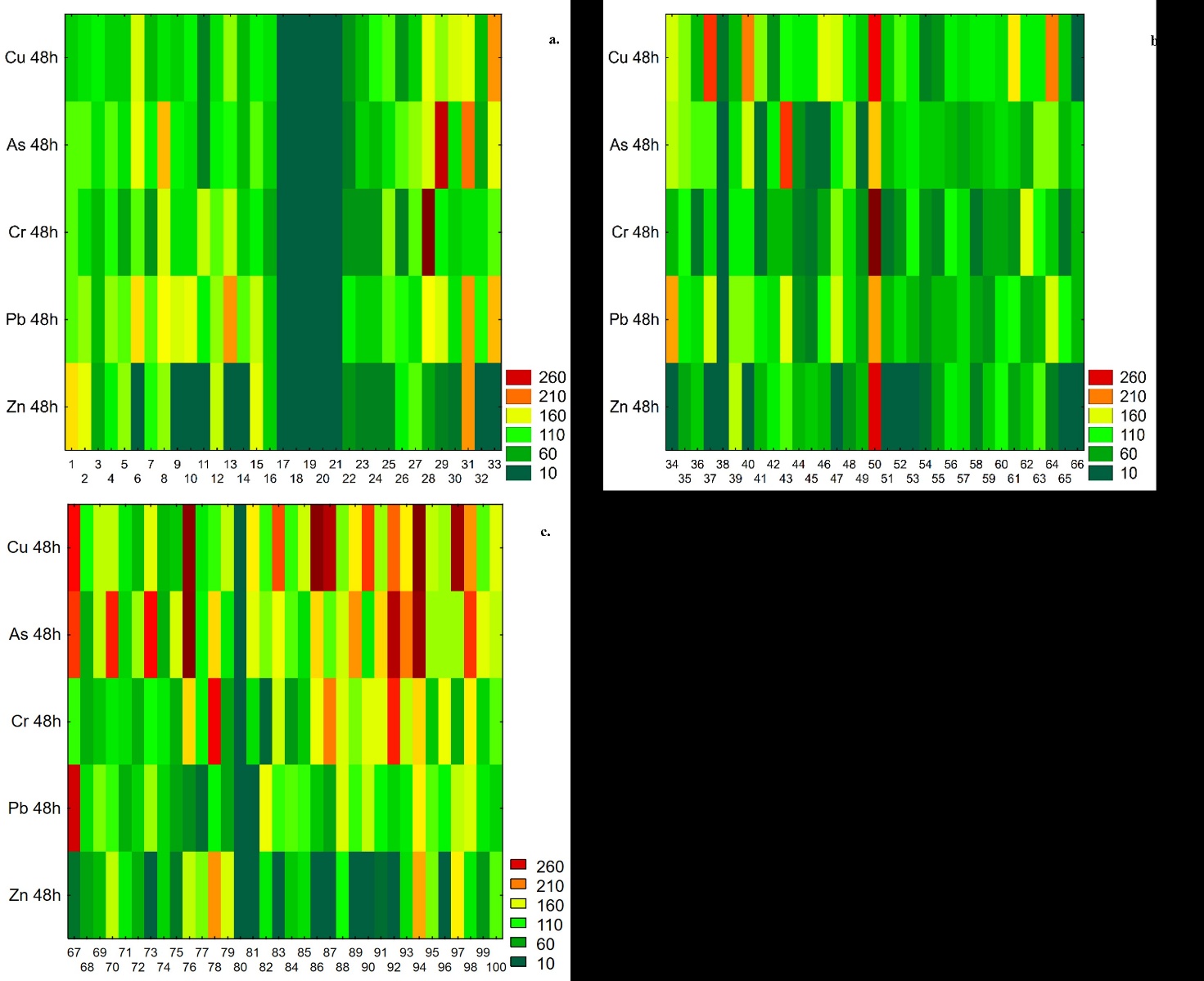

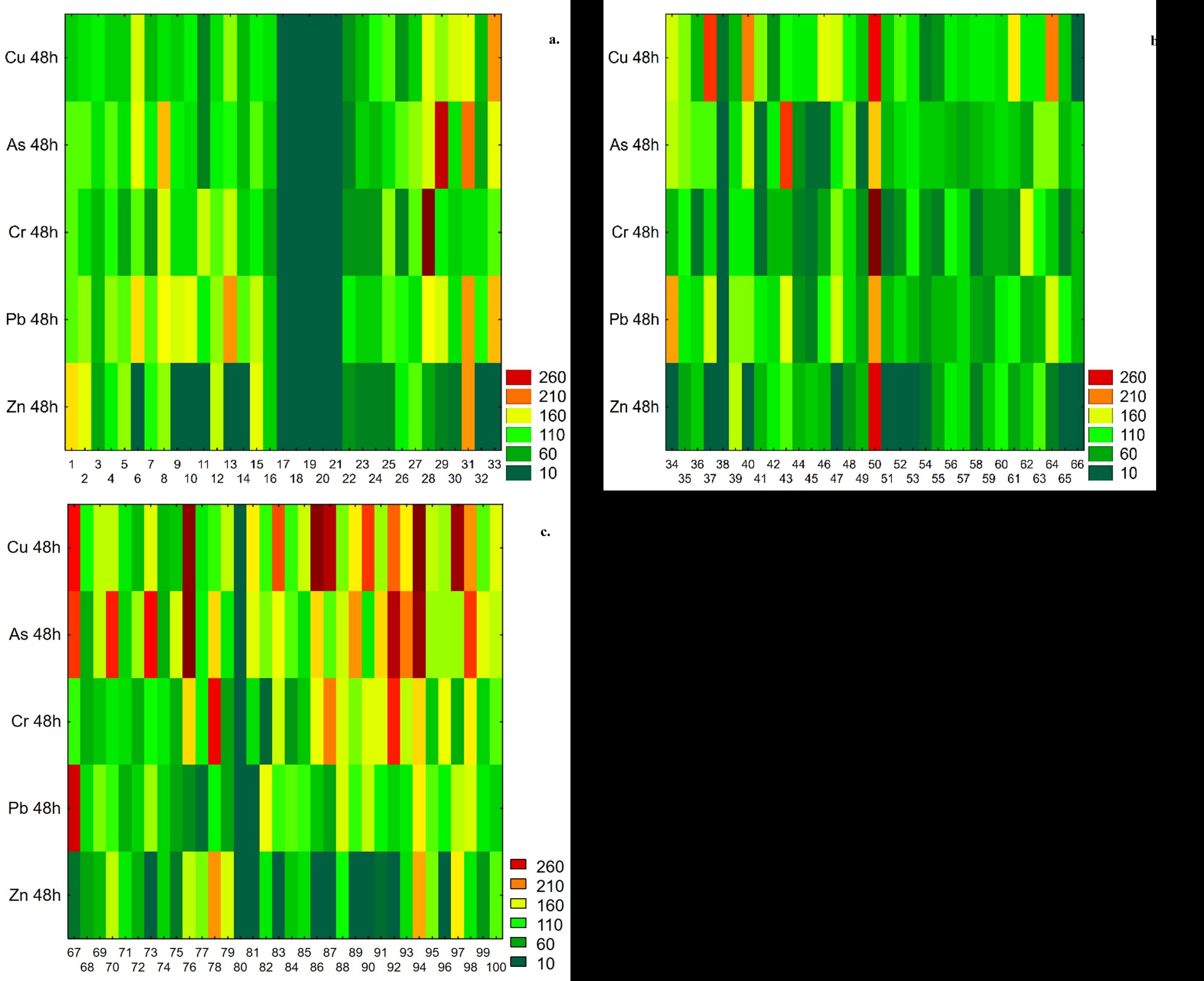


**c**

**b**

**a**

**Supplementary Figure S1.** Heatmap showing Growth Index (GI) (%) values of *Pseudomonas* isolates under osmotic stress induced by PEG at concentrations of 5%, 10%, 15%, and 20%. Isolates are grouped in numerical order and displayed along the x-axis (**4A** from strain 1 to 33, **4B**: from strain 34 to 66, **4C**: from strain 67 to 100). GI values are color-coded from dark green (strong inhibition, GI = –100) to dark red (slightly enhanced growth, GI = +60).
